# Supplementary material for: Hyper-cores promote localization and efficient seeding in higher-order processes
Source: Nat Commun. 2023 Oct 6;14:6223. doi: 10.1038/s41467-023-41887-2 (PMC10558485; doi:10.1038/s41467-023-41887-2)
Supplement: Supplementary file 1 — Supplementary Information [file 41467_2023_41887_MOESM1_ESM.pdf]

Supplementary Information for "Hyper-cores promote localization and efficient seeding in higher-order processes"

Marco Mancastroppa,[1] Iacopo Iacopini,[2,3] Giovanni Petri,[2,4] and Alain Barrat[1]

[1]*Aix Marseille Univ, Université de Toulon, CNRS, CPT, Turing Center for Living Systems, Marseille, France*

[2]*Network Science Institute, Northeastern University London, London, E1W 1LP, United Kingdom*

[3]*Department of Network and Data Science, Central European University, 1100 Vienna, Austria*

[4]*CENTAI, Corso Inghilterra 3, 10138 Turin, Italy*

In this Supplementary Information we present the same results as in the main text for all the considered data sets and also further results. In Supplementary Note I we present in detail some of the statistical properties of the data sets and of the static hypergraphs considered. In Supplementary Note II we present the results of the (k, m)-core decomposition, showing how the (k, m)-cores and (k, m)-shells are populated as a function of k and m, the functional form of the m-shell index $C_m(i)$ for some nodes, the distributions of the size-independent and frequency-based hyper-coreness centralities, k-coreness and s-coreness centralities and their correlations. In Supplementary Note III we consider the randomized realizations of the empirical hypergraphs, obtained through the shuffling procedure described in the Methods of the main text [1, 2]: we compare the (k, m)-core decomposition of the randomized realizations to that of the empirical hypergraphs, by investigating their differences in the (k, m)-cores population as a function of k and m and in the functional form of the maximum connectivity value k^m_{max}. In Supplementary Note IV we present the results of the higher-order non-linear contagion process [3], both in the SIS and SIR formulation, also comparing the performance of different centralities in identifying central nodes for the dynamic processes. In Supplementary Note V we introduce in details the threshold higher-order process [4], its numerical implementation and its results in relation to the hyper-cores, both in the SIS and SIR formulation, as done for the higher-order non-linear contagion model. In Supplementary Note VI, the results of the higher-order naming-game process [5] are presented for both the union and the unanimity rules. Finally in Supplementary Note VII, we report a sketch of the hyper-core decomposition procedure.

I. SUPPLEMENTARY NOTE 1: PROPERTIES OF THE DATA SETS

The considered data sets describe interactions in several environments, mediated by different mechanisms, and thus they differ in their fundamental statistical properties. This is summarized in Supplementary Table I and Supplementary Fig. 1: the number of nodes and hyperedges vary among the data sets considered, the distribution $\Psi(m)$ of the hyperedge sizes m, the range of their sizes $m \in [2, M]$ and the average hyperedge size $\langle m \rangle$ are different among the data sets.

data set	N	E	M	$\langle m \rangle$
LH10	76	1 102	7	3.4
Thiers13	327	4 795	7	3.1
InVS15	217	3 279	10	2.8
SFHH	403	6 398	10	2.7
LyonSchool	242	10 848	10	4.0
Mid1	591	61 521	13	3.9
Elem1	339	20 940	16	4.7
email-EU	979	24 399	25	3.5
congress-bills	1 718	83 105	25	8.8
senate-committees	282	302	31	17.6

data set	N	E	M	$\langle m \rangle$
email-Enron	143	1 459	37	3.1
house-committees	1 290	335	82	35.3
music-review	1 106	686	83	15.3
senate-bills	294	21 721	99	9.9
algebra-questions	423	980	107	7.6
geometry-questions	580	888	230	13.0
M_PL_015_ins	666	127	124	22.9
M_PL_015_pl	130	401	104	6.6
M_PL_062_ins	1 044	456	58	33.5
M_PL_062_pl	456	866	157	17.4

Supplementary Table I: **Some properties of the data sets.** The tables give: the number of nodes N, the number of hyperedges E, the maximum size of the hyperedges M and the average size of the hyperedges $\langle m \rangle$.

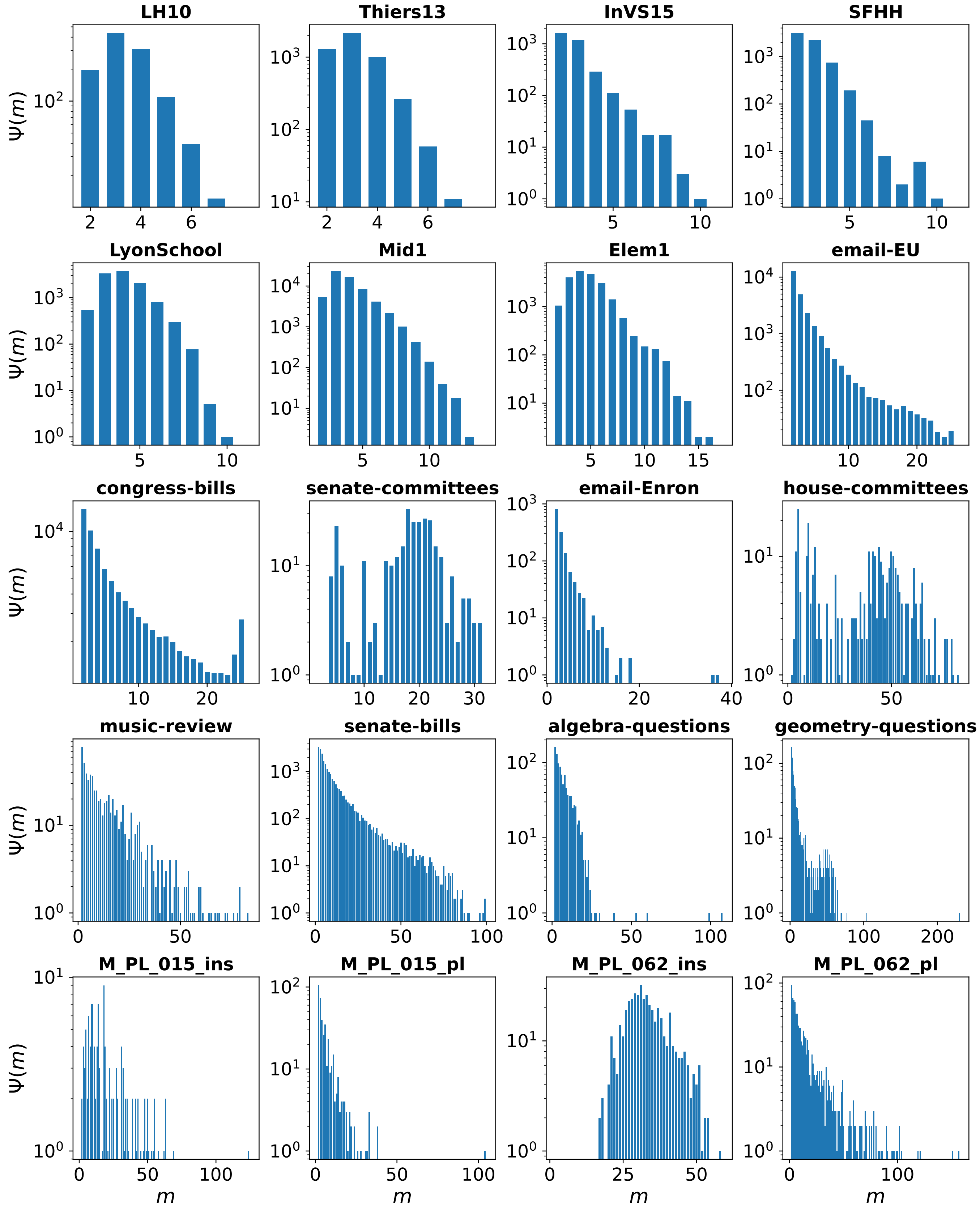

Supplementary Figure 1: **Hyperedge size distribution.** We show the hyperedge size distribution $\Psi(m)$, i.e. the number of hyperedges of size m, for all the data sets.

II. SUPPLEMENTARY NOTE 2: HYPER-CORE DECOMPOSITION

In this Supplementary Note we present the results of the (k, m)-core decomposition on all the considered data sets: we show the (k, m)-cores and (k, m)-shells relative population size as a function of k and m (Supplementary Figs. 2-4), the functional form of the m-shell index $C_m(i)$ for some specific nodes (Supplementary Fig. 5), the distributions of the nodes size-independent and frequency-based hyper-coreness, k-coreness and s-coreness centralities (Supplementary Figs. 6-9) and their correlations (Supplementary Figs. 10-12).

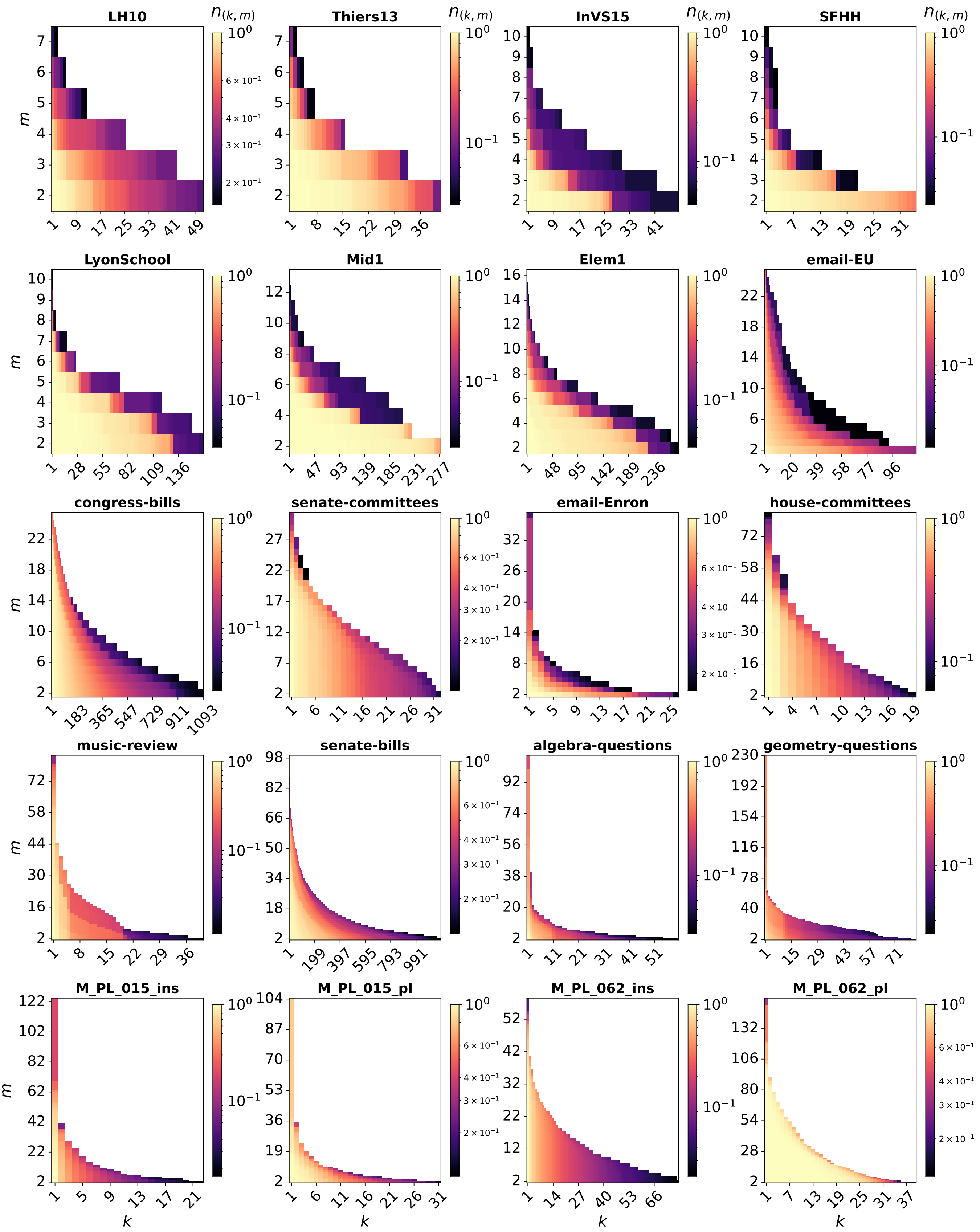

Supplementary Figure 2: **Hyper-core decomposition I.** All panels show colormaps giving the relative size $n_{(k,m)}$ (number of nodes in the hyper-core, divided by the total number of nodes N) of the (k, m)-hyper-core as a function of m and k (white regions correspond to $n_{(k,m)} = 0$).

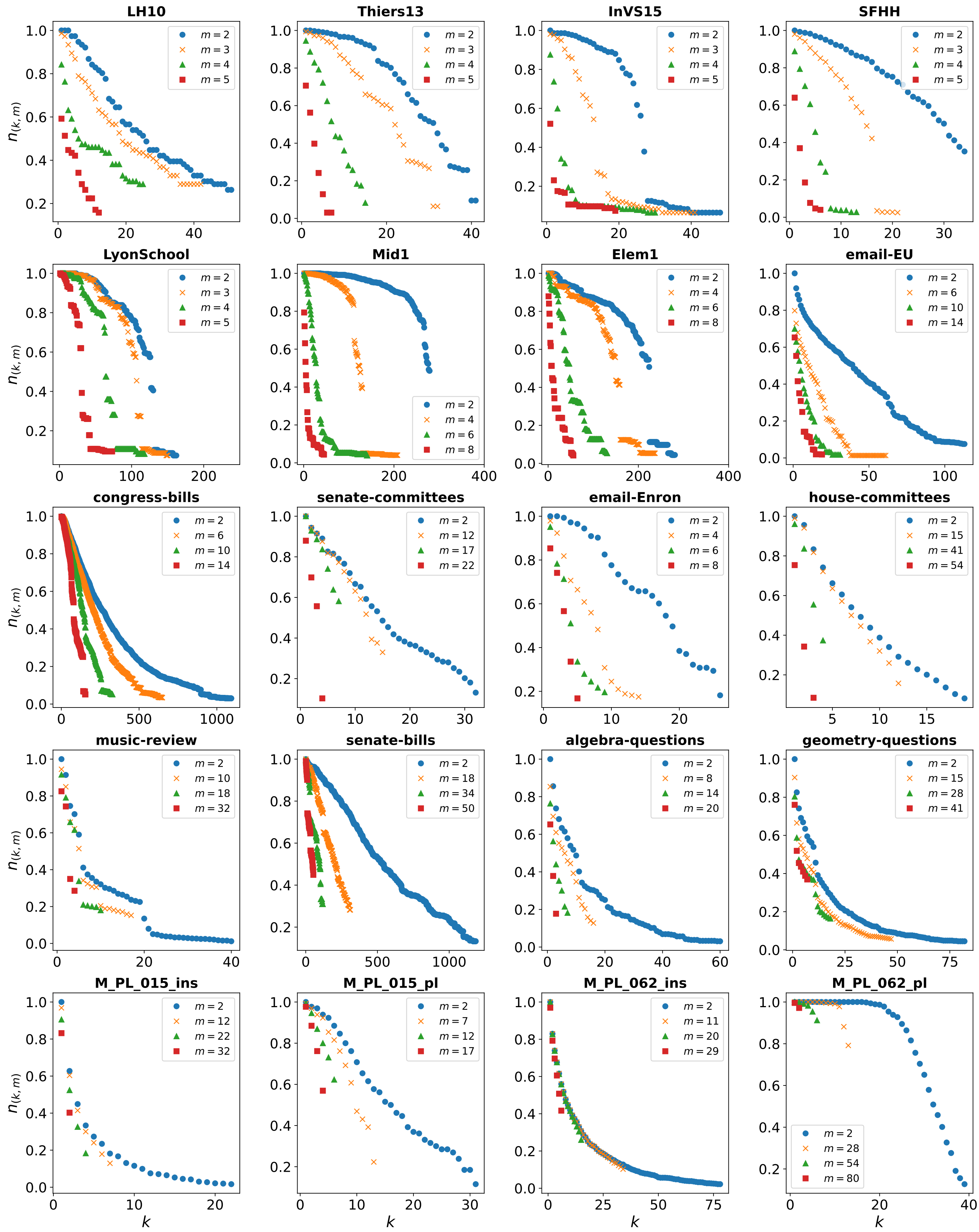

Supplementary Figure 3: **Hyper-core decomposition II.** All panels show the relative size $n_{(k,m)}$ (number of nodes in the hyper-core, divided by the total number of nodes N) of the (k, m)-hyper-core as a function of k for fixed values of m.

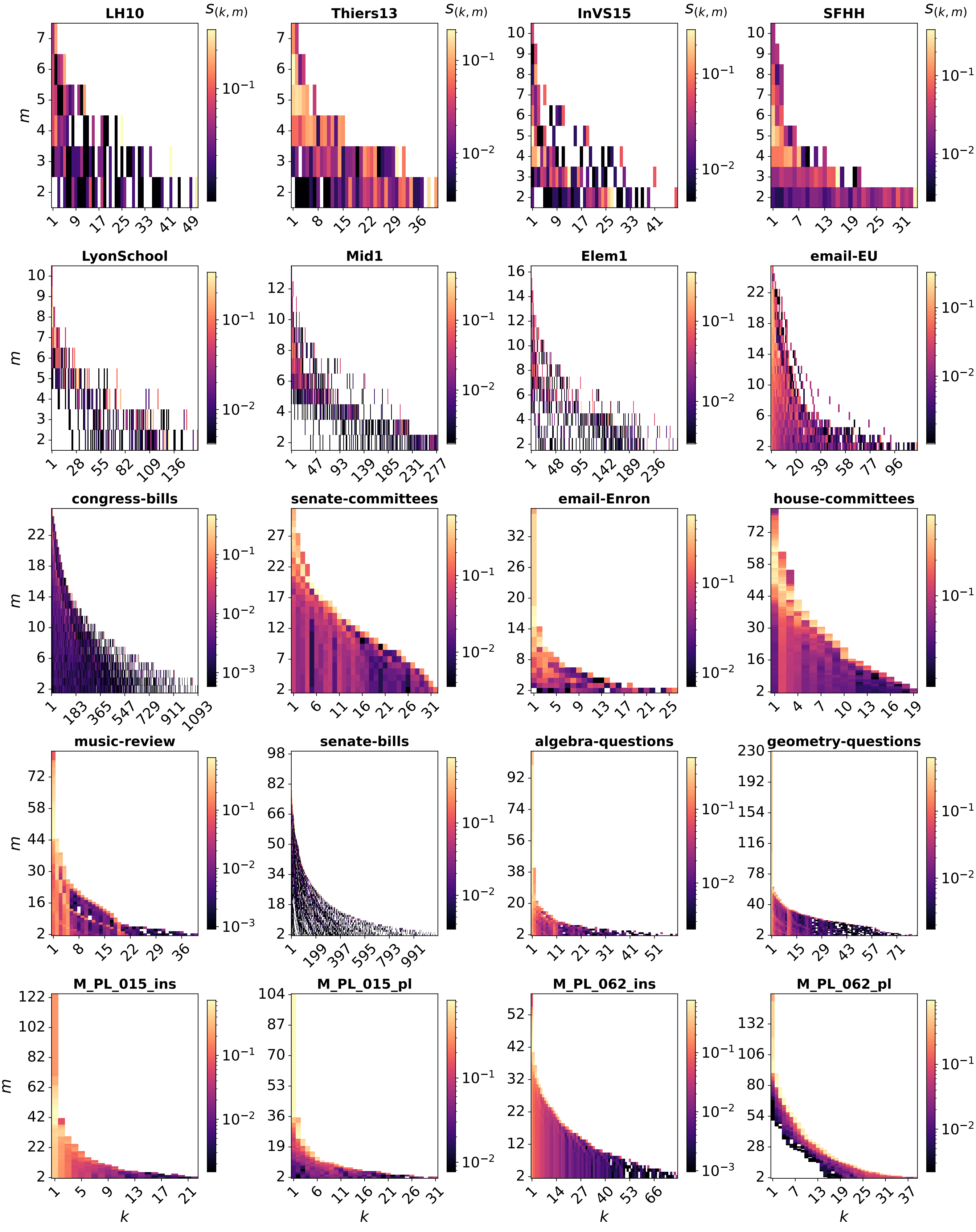

Supplementary Figure 4: (k, m)**-shells.** All panels show colormaps giving the relative size $s_{(k,m)}$ (number of nodes in the hyper-shell, divided by the total number of nodes N) of the (k, m)-shell as a function of m and k (white regions correspond to $s_{(k,m)} = 0$).

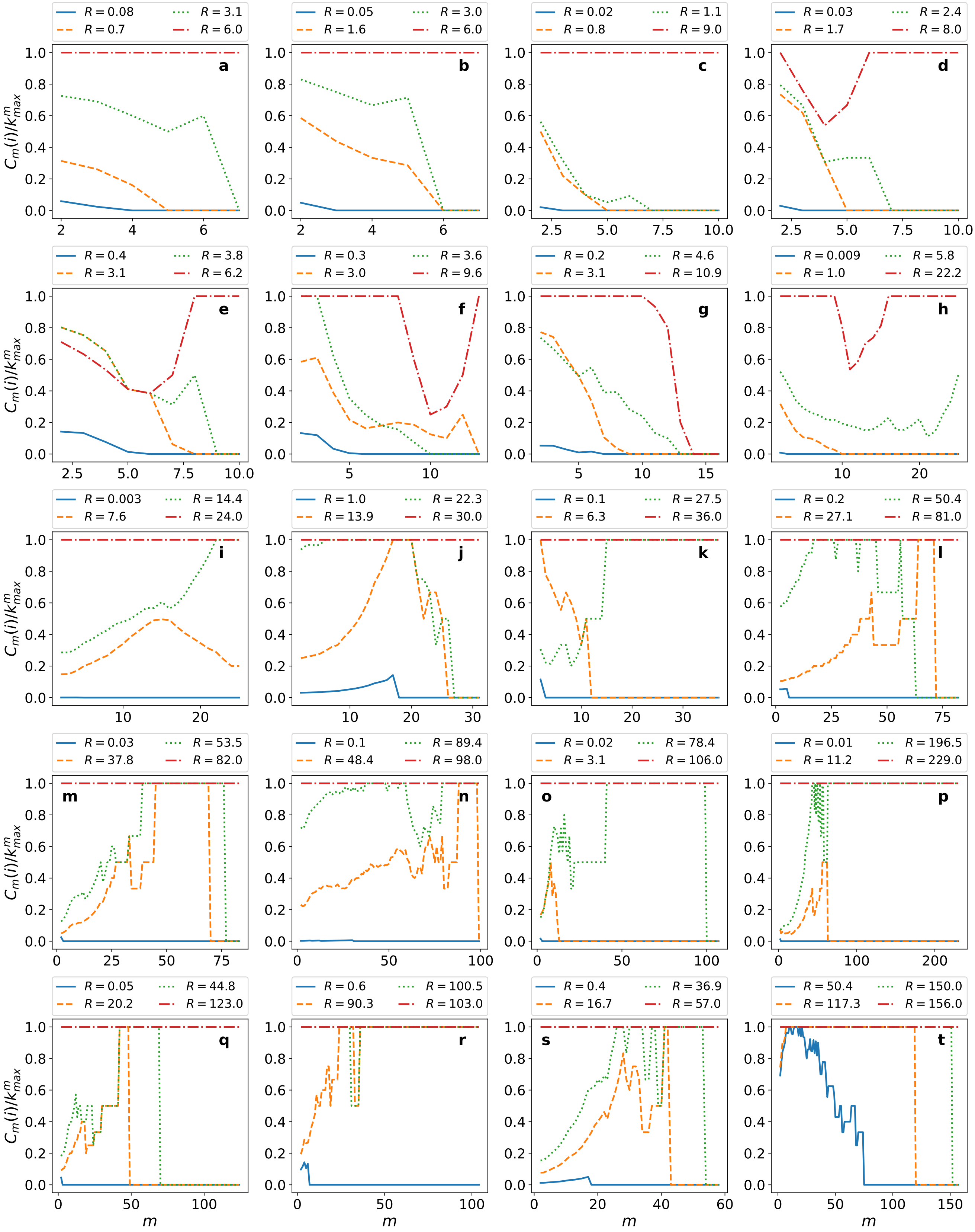

Supplementary Figure 5: **m-shell index.** All panels show the normalized m-shell index function $C_m(i)/k^m_{max}$ as a function of m for four nodes: one node is selected randomly among the nodes in the class with highest size-independent hyper-coreness R; one node is selected randomly among the nodes in the class with smallest size-independent hyper-coreness R; the two remaining nodes are selected from intermediate hyper-coreness classes, so that the positions in the hyper-coreness ranking of the four nodes are equispaced. The following data sets are considered: LH10 (panel **a**), Thiers13 (panel **b**), InVS15 (panel **c**), SFHH (panel **d**), LyonSchool (panel **e**), Mid1 (panel **f**), Elem1 (panel **g**), email-EU (panel **h**), congress-bills (panel **i**), senate-committees (panel **j**), email-Enron (panel **k**), house-committees (panel **l**), music-review (panel **m**), senate-bills (panel **n**), algebra-questions (panel **o**), geometry-questions (panel **p**), M_PL_015_ins (panel **q**), M_PL_015_pl (panel **r**), M_PL_062_ins (panel **s**) and M_PL_062_pl (panel **t**).

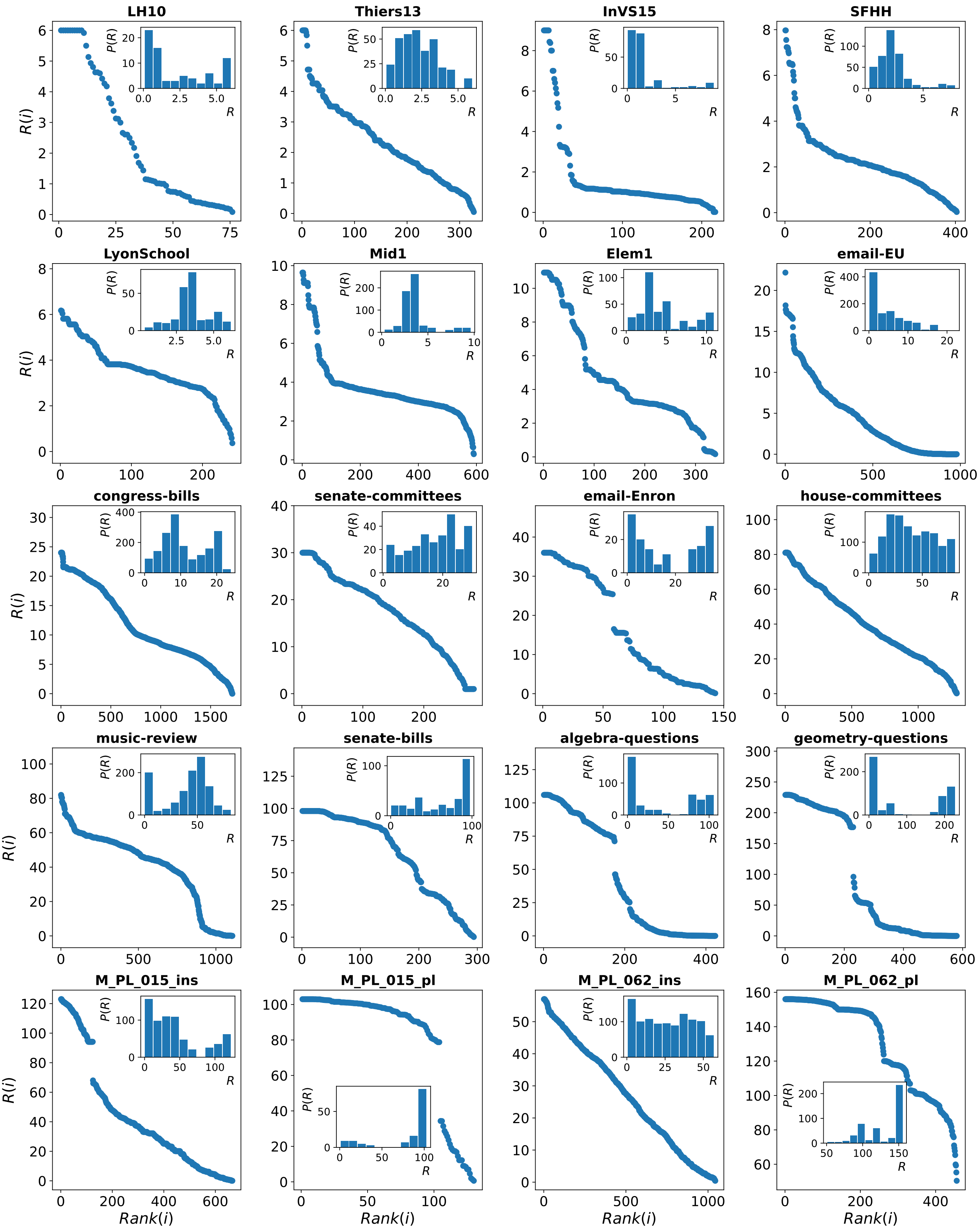

Supplementary Figure 6: **Size-independent hyper-coreness centrality.** In all panels the size-independent hyper-coreness $R(i) = \sum_{m=2}^{M} C_m(i)/k_{max}^m$ is plotted as a function of the corresponding node rank: the insets show the distribution $P(R)$ of the size-independent hyper-coreness.

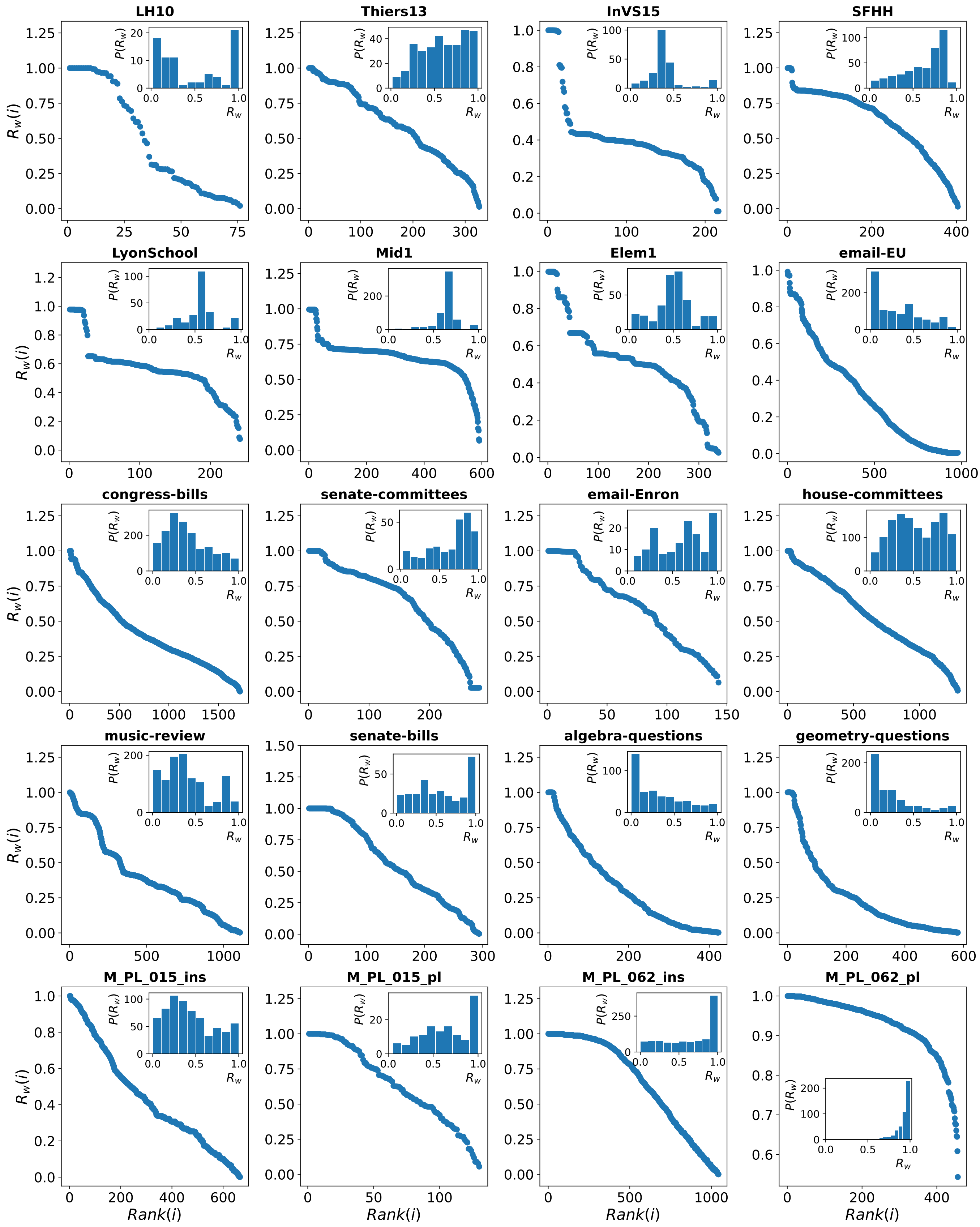

Supplementary Figure 7: **Frequency-based hyper-coreness centrality.** In all panels the frequency-based hyper-coreness $R_w(i) = \sum_{m=2}^{M} \Psi(m) C_m(i)/k_{max}^m$ is plotted as a function of the corresponding node rank: the insets show the distribution $P(R_w)$ of the frequency-based hyper-coreness.

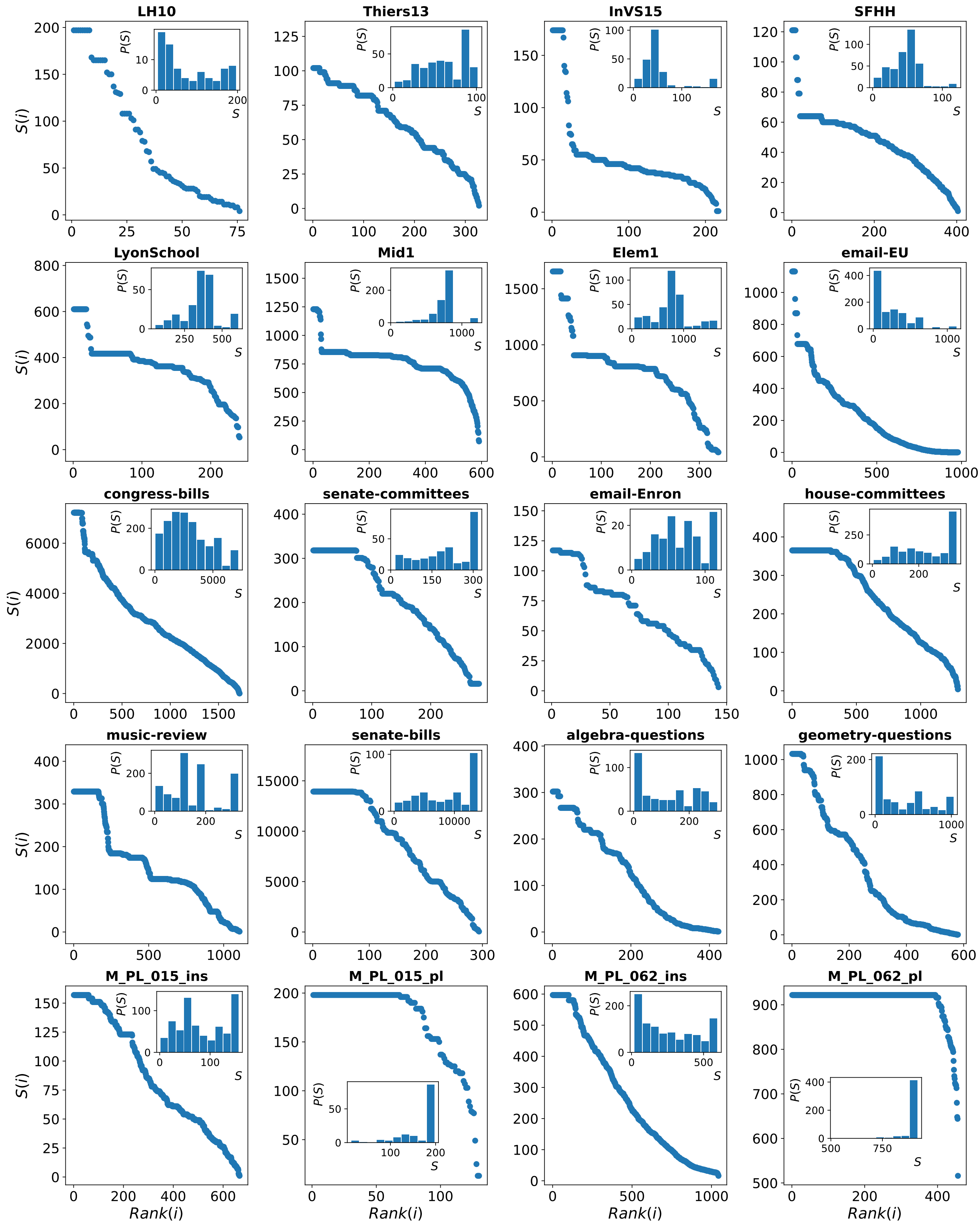

Supplementary Figure 8: **s-coreness centrality.** In all panels the s-coreness $S(i)$ is plotted as a function of the corresponding node rank: the insets give the distribution $P(S)$ of the s-coreness.

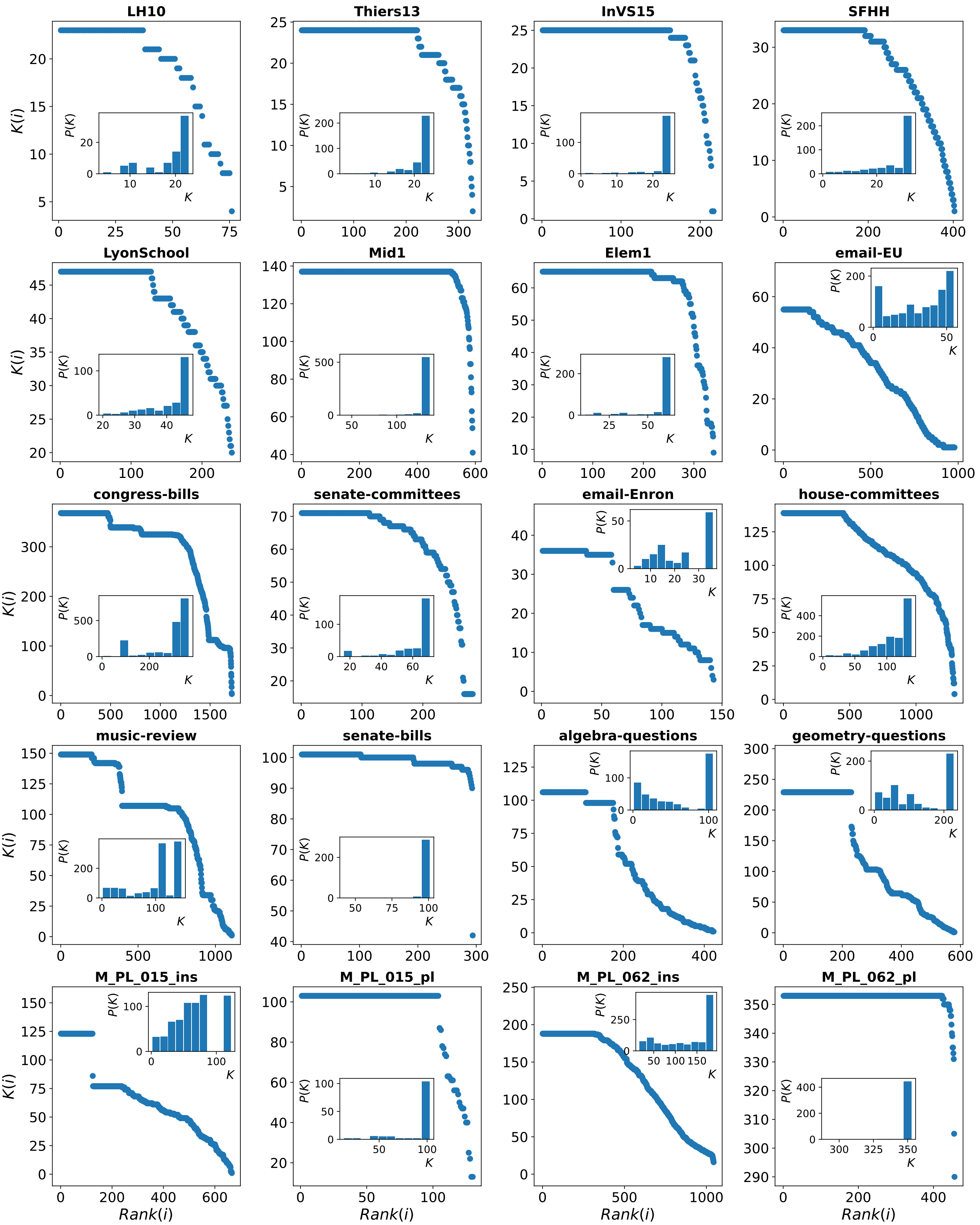

Supplementary Figure 9: **k-coreness centrality.** In all panels the k-coreness $K(i)$ is plotted as a function of the corresponding node rank: the insets give the distribution $P(K)$ of the k-coreness.

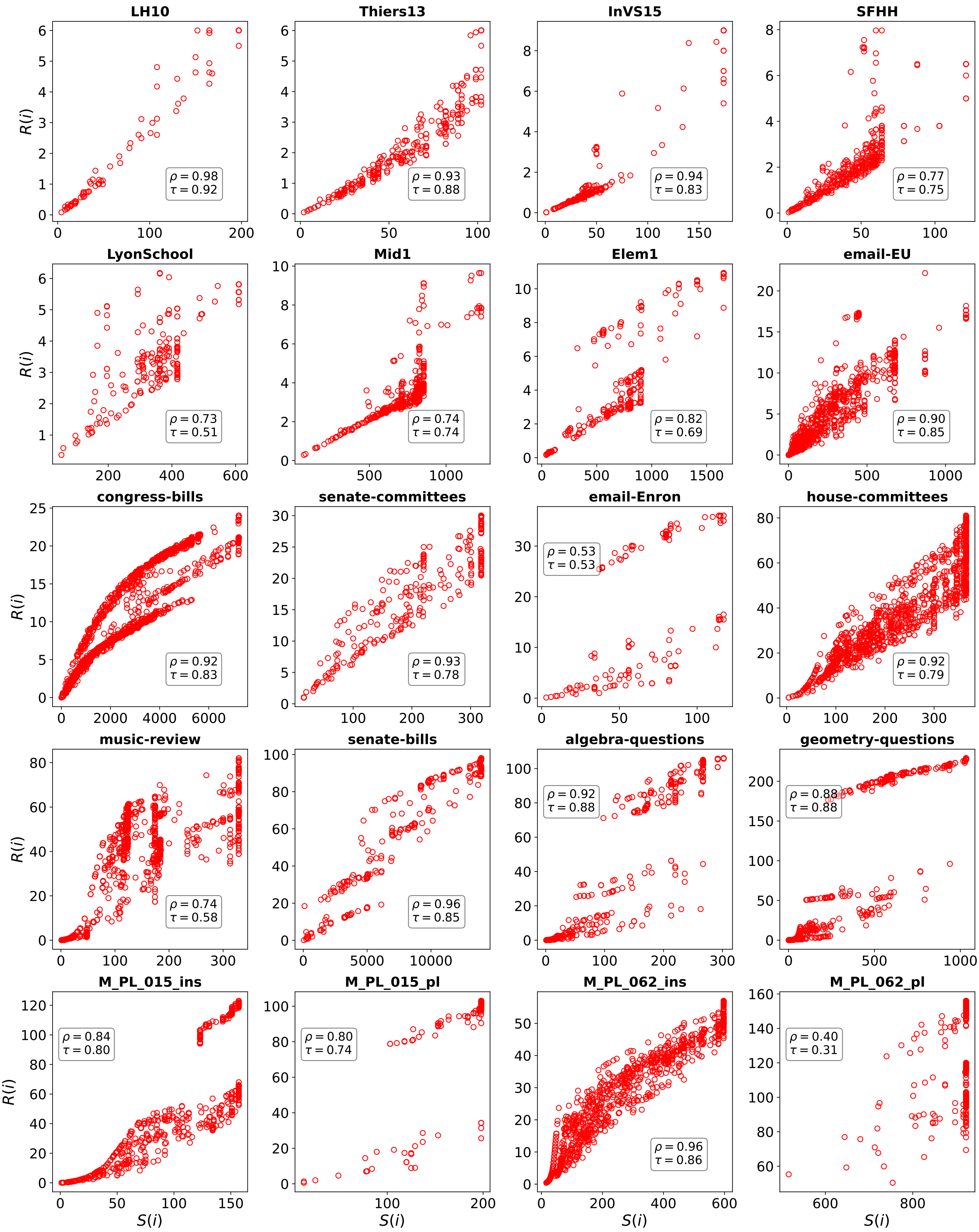

Supplementary Figure 10: **Hyper-coreness vs. s-coreness centralities.** All panels show scatterplots of the size-independent hyper-coreness $R(i)$ vs. the s-coreness $S(i)$ for all nodes: the text-box reports the Pearson correlation coefficient ρ of $R(i)$ and $S(i)$ and the Kendall's τ coefficient of the corresponding node rankings (in all cases the p-value for both the coefficients is $p \ll 0.001$).

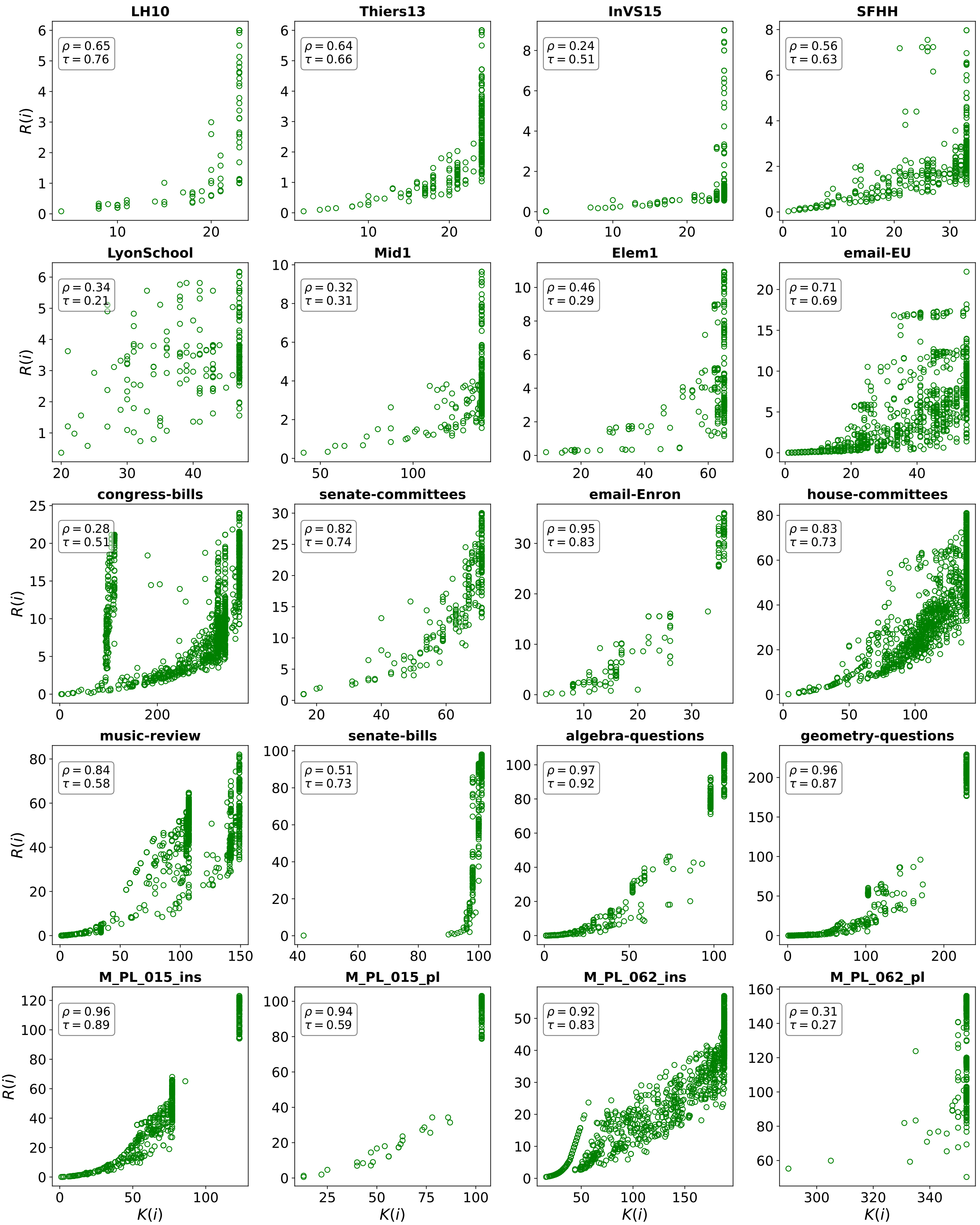

Supplementary Figure 11: **Hyper-coreness vs. k-coreness centralities.** All panels show scatterplots of the size-independent hyper-coreness $R(i)$ vs. the k-coreness $K(i)$ for all nodes: the text-box reports the Pearson correlation coefficient ρ of $R(i)$ and $K(i)$ and the Kendall's τ coefficient of the corresponding node rankings (in all cases the p-value for both the coefficients is $p \ll 0.001$).

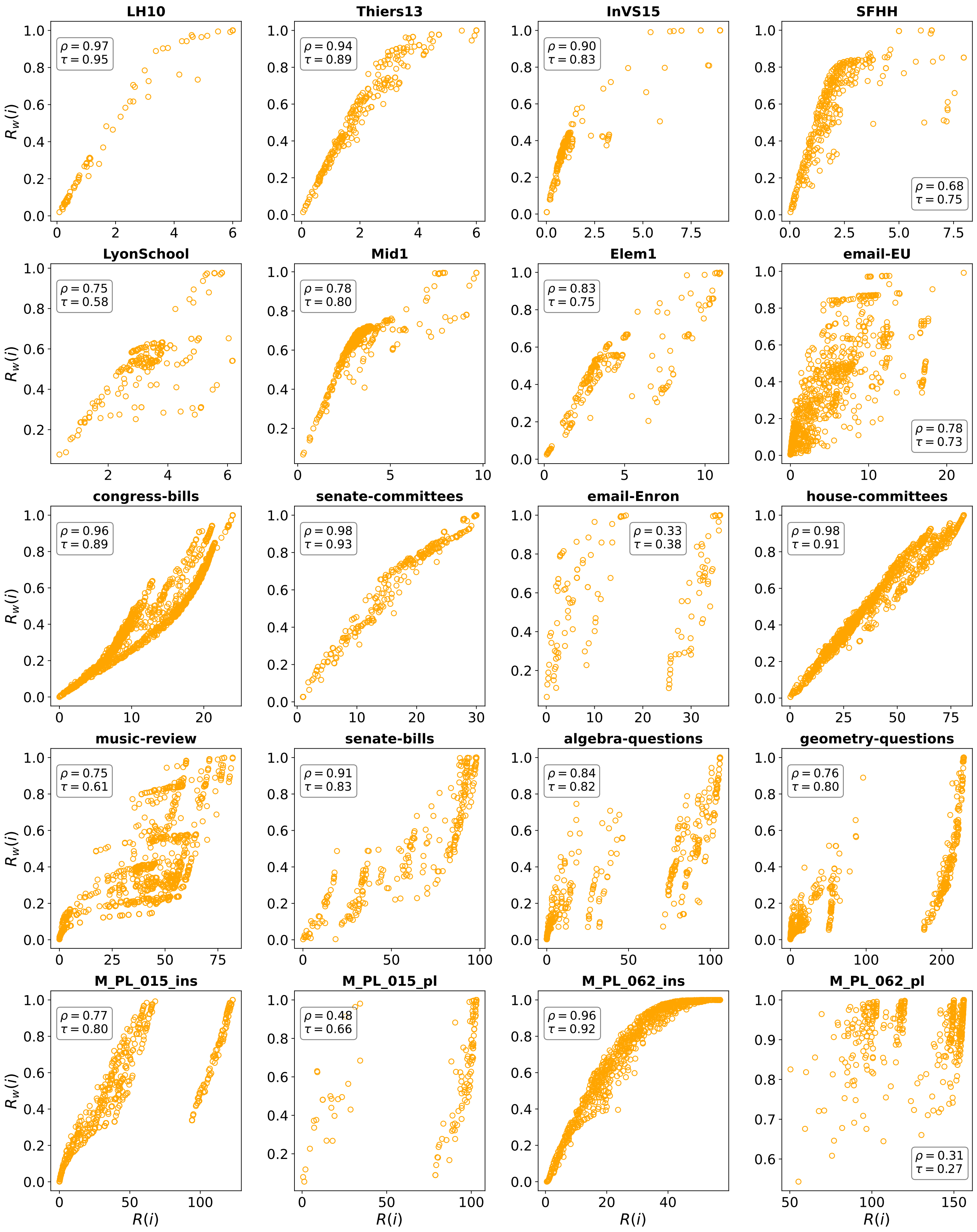

Supplementary Figure 12: **Frequency-based hyper-coreness vs. size-independent hyper-coreness centralities.** All panels show scatterplots of the frequency-based hyper-coreness $R_w(i)$ vs. the size-independent hyper-coreness $R(i)$ for all nodes: the text-box reports the Pearson correlation coefficient ρ of $R_w(i)$ and $R(i)$ and the Kendall's τ coefficient of the corresponding node rankings (in all cases the p-value for both the coefficients is $p \ll 0.001$).

III. SUPPLEMENTARY NOTE 3: DIFFERENCES BETWEEN EMPIRICAL HYPERGRAPHS AND THEIR RANDOMIZED REALIZATIONS

In this Supplementary Note we consider the randomized realizations of the empirical hypergraphs for all the considered data sets: the randomized realizations are obtained through the shuffling procedure described in the Methods of the main text [1, 2]. We estimate how the (k, m)-core decomposition of the randomized realizations differs from that of the empirical hypergraphs, by investigating how the (k, m)-cores are populated as a function of k and m (Supplementary Figs. 13-14) and the functional form of the maximum connectivity value k^m_{max} (Supplementary Fig. 15).

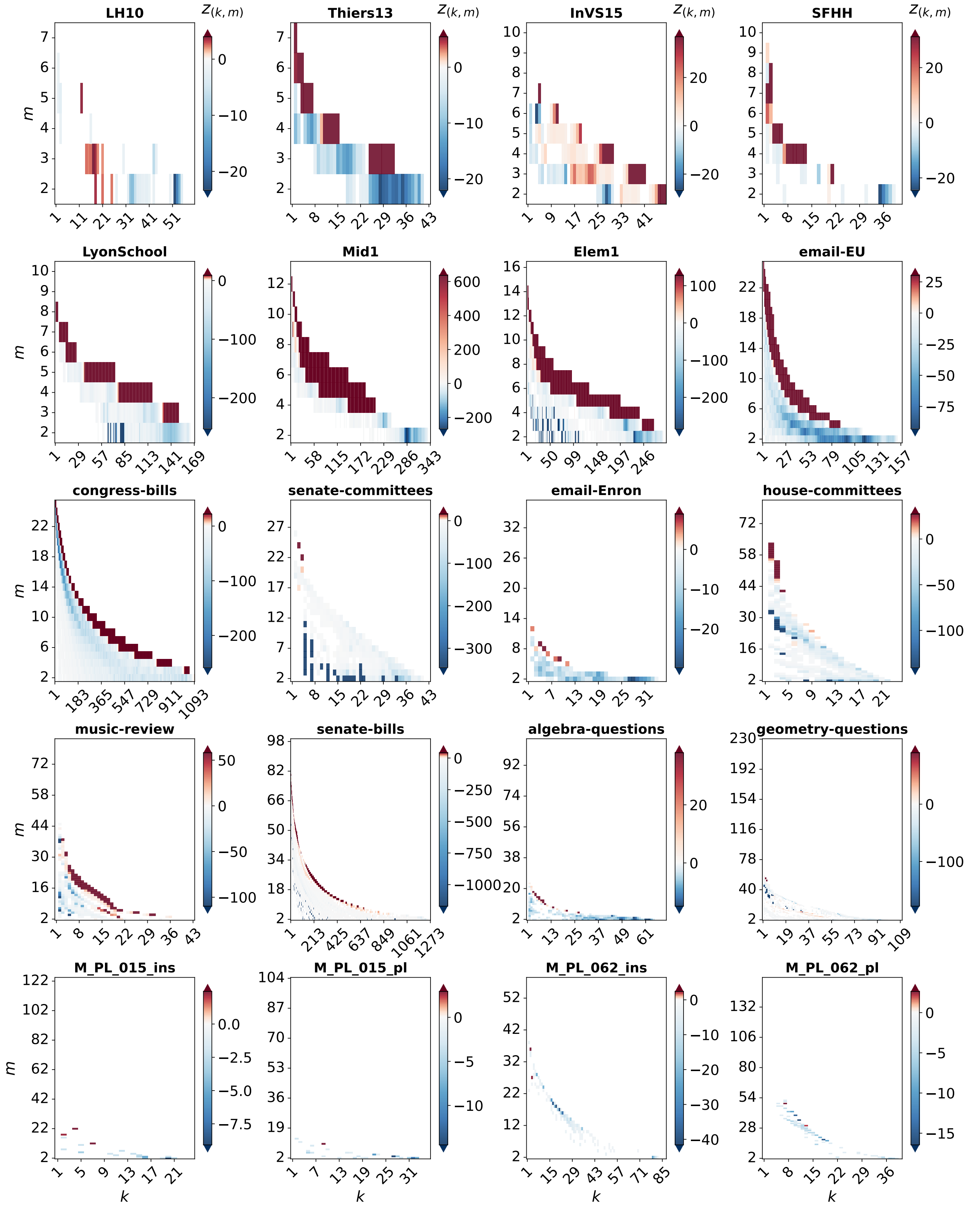

Supplementary Figure 13: **Randomized hypergraphs I.** All panels show colormaps giving the z-score $z_{(k,m)} = (n_{(k,m)} - \mu_{(k,m)})/\sigma_{(k,m)}$ as a function of m and k: $n_{(k,m)}$ is the fraction of population in the (k, m)-core of the empirical hypergraph; $\mu_{(k,m)}$ and $\sigma_{(k,m)}$ are respectively the mean and standard deviation of the fraction of population in the (k, m)-cores of the corresponding shuffled realizations. In all panels we consider 10^3 random realizations of the empirical hypergraphs; only values of $z_{(k,m)} \leq -1.96$ and $z_{(k,m)} \geq 1.96$ are shown, while values $z_{(k,m)} \in (-1.96, 1.96)$ are marked in white.

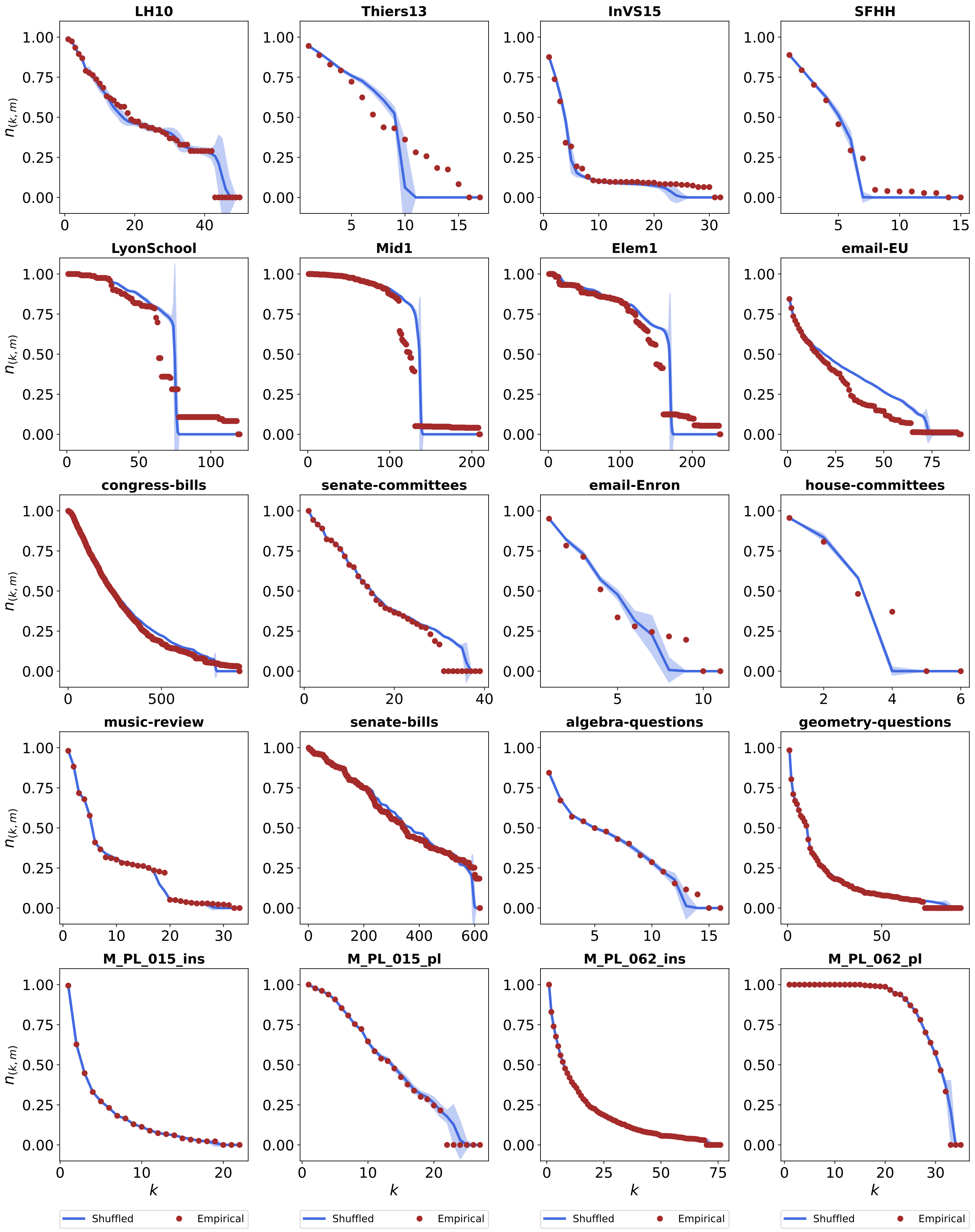

Supplementary Figure 14: **Randomized hypergraphs II.** All panels show the relative population size $n_{(k,m)}$ of the (k, m)-hyper-core as a function of k for a fixed m value, for both the empirical hypergraph (red dots) and the corresponding randomized realizations, by showing the average relative size of the (k, m)-hyper-core (blue solid line - the blue shaded area indicates values which would correspond to a z-score $z_{(k,m)} \in [-1.96, 1.96]$). In all panels we consider 10^3 random realizations of the empirical hypergraphs and we fix $m = 4$, apart for LH10 where $m = 3$, email-Enron where $m = 6$, house-committees where $m = 42$, senate-bills and algebra-questions where $m = 9$.

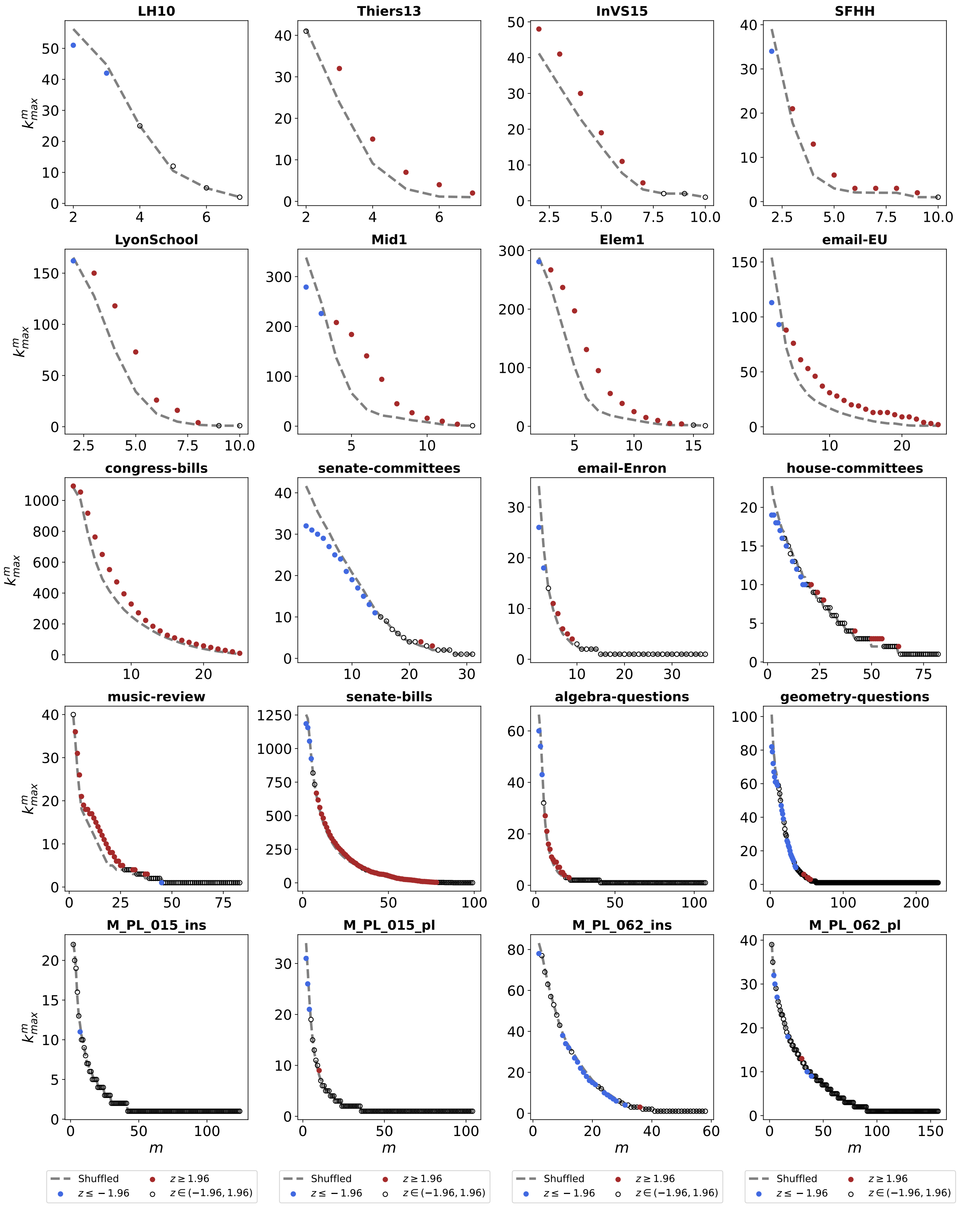

Supplementary Figure 15: **Randomized hypergraphs - k^m_{max}.** All panels show the maximum connectivity value k^m_{max}, i.e. the maximum k such that the (k, m)-shell is not empty, as a function of m: the grey dashed line correspond to $\langle k^m_{max} \rangle$ averaged over the shuffled realizations of the empirical hypergraph; the dots correspond to the k^m_{max} values in the empirical hypergraph (red dots if the corresponding z-score $z_m = (k^m_{max} - \langle k^m_{max} \rangle)/\sigma_m$ is $z_m \geq 1.96$; blue dots if $z_m \leq -1.96$; empty dots if $z_m \in (-1.96, 1.96)$). In all panels we consider 10^3 random realizations of the empirical hypergraphs.

IV. SUPPLEMENTARY NOTE 4: HIGHER-ORDER NON-LINEAR CONTAGION PROCESS

In this Supplementary Note we present the results of the higher-order non-linear contagion process [3] on empirical hypergraphs, both in the SIS (Supplementary Figs. 16-20) and SIR formulation (Supplementary Figs. 21-25), also comparing the performance of different centralities in identifying central nodes for the dynamic processes (Supplementary Figs. 19-20 and Supplementary Figs. 24-25).

**data set**	ν	λ
LH10	4.0	5×10^{-4}
Thiers13	3.0	5×10^{-4}
InVS15	4.0	5×10^{-4}
SFHH	4.0	5×10^{-4}
LyonSchool	2.0	5×10^{-4}
Mid1	3.0	5×10^{-5}
Elem1	1.5	5×10^{-5}
email-EU	2.5	5×10^{-5}
congress-bills	2.0	5×10^{-6}
senate-committees	1.25	5×10^{-4}

**data set**	ν	λ
email-Enron	2.0	5×10^{-4}
house-committees	1.25	5×10^{-4}
music-review	1.25	5×10^{-4}
senate-bills	1.5	5×10^{-6}
algebra-questions	1.25	5×10^{-4}
geometry-questions	1.5	5×10^{-5}
M_PL_015_ins	1.25	5×10^{-4}
M_PL_015_pl	1.25	5×10^{-4}
M_PL_062_ins	1.25	5×10^{-4}
M_PL_062_pl	1.25	5×10^{-4}

Supplementary Table II: **Parameters for Supplementary Figs. 16-20.** The tables summarize the parameters of the higher-order non-linear SIS contagion process considered for each data set in Supplementary Figs. 16-20.

**data set**	ν	λ
LH10	1.5	0.010
Thiers13	4.0	0.001
InVS15	4.0	0.001
SFHH	4.0	0.010
LyonSchool	4.0	0.001
Mid1	4.0	5×10^{-5}
Elem1	4.0	10^{-4}
email-EU	4.0	5×10^{-5}
congress-bills	1.5	5×10^{-5}
senate-committees	4.0	10^{-4}

**data set**	ν	λ
email-Enron	4.0	5×10^{-4}
house-committees	4.0	5×10^{-5}
music-review	3.0	5×10^{-4}
senate-bills	4.0	5×10^{-5}
algebra-questions	4.0	0.001
geometry-questions	4.0	5×10^{-4}
M_PL_015_ins	1.25	5×10^{-4}
M_PL_015_pl	2.0	5×10^{-4}
M_PL_062_ins	4.0	5×10^{-5}
M_PL_062_pl	4.0	5×10^{-5}

Supplementary Table III: **Parameters for Supplementary Figs. 21-25.** The tables summarize the parameters of the higher-order non-linear SIR contagion process considered for each data sets in Supplementary Figs. 21-25.

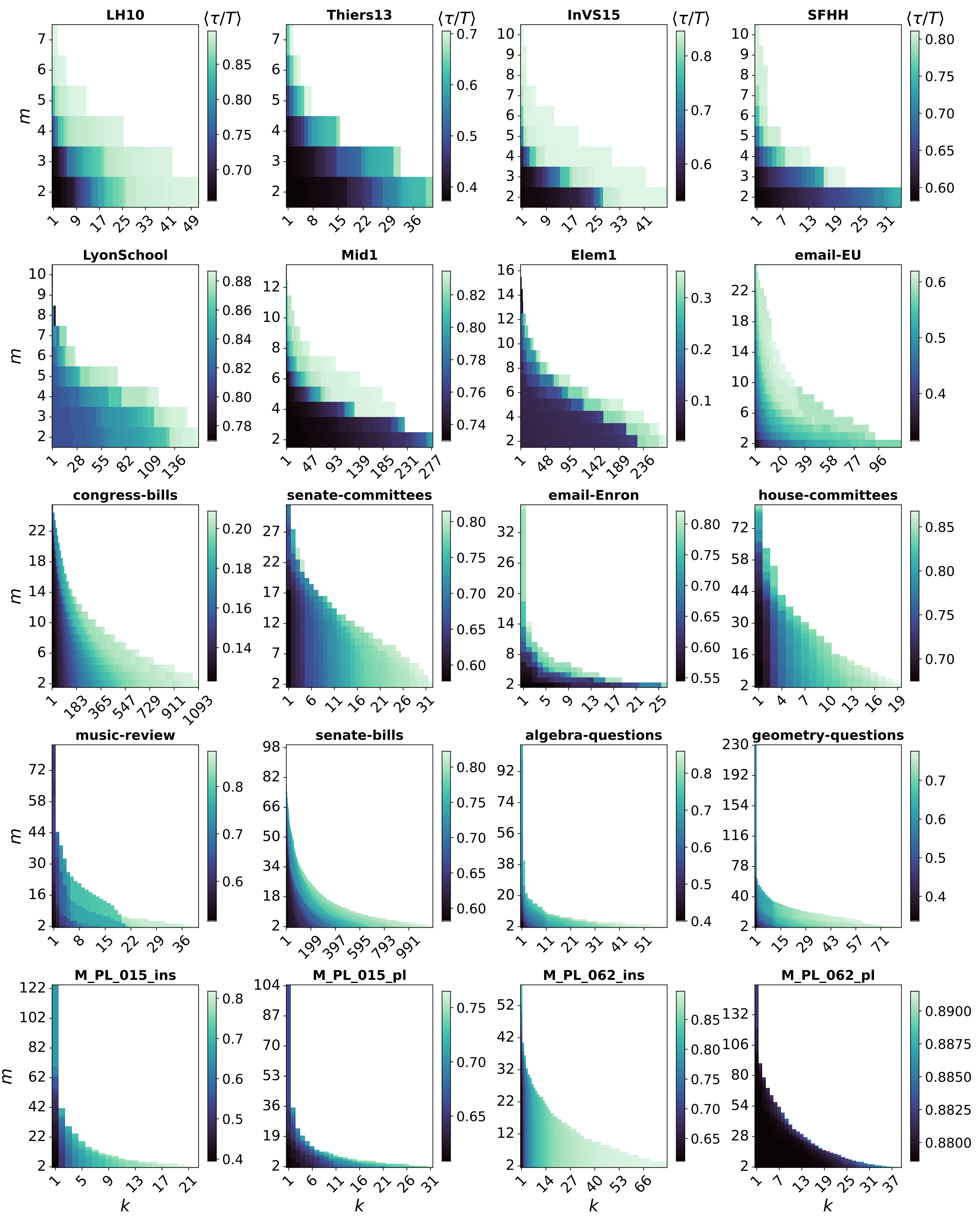

Supplementary Figure 16: **Higher-order non-linear contagion process - SIS model - I.** All panels give, as a heat-map as a function of k and m, the average fraction $\langle \tau/T \rangle$ of time being infected in the SIS steady state averaged over the nodes of the (k, m)-hyper-core. All results are obtained by averaging the results of 10^3 numerical simulations, with a single random seed of infection and with an observation window $T = 10^3$. The (λ, ν) values considered for each data set are reported in Supplementary Table II and in all panels $\mu = 0.1$.

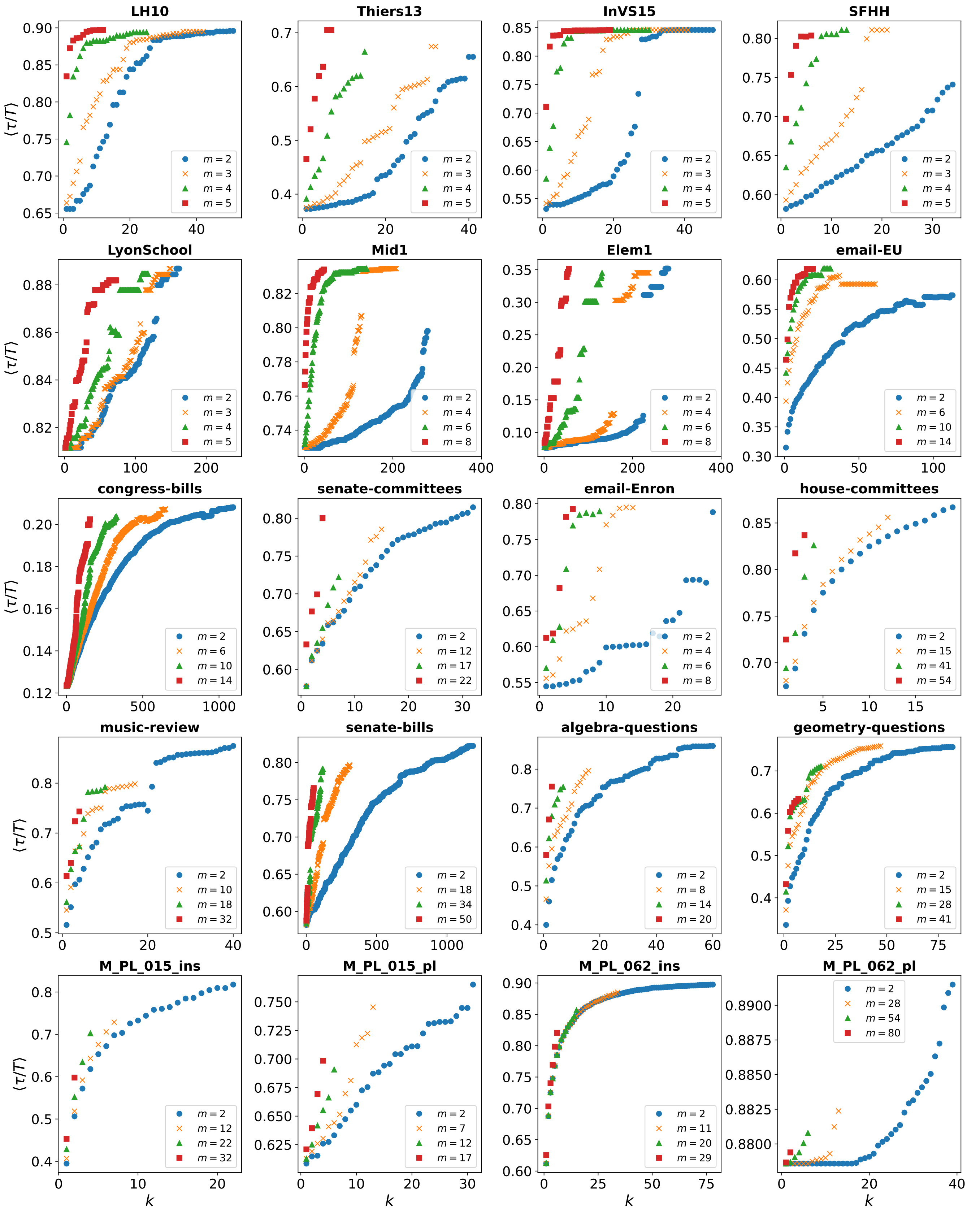

Supplementary Figure 17: **Higher-order non-linear contagion process - SIS model - II.** In all panels the average fraction $\langle \tau/T \rangle$ of time being infected in the SIS steady state averaged over the nodes of the (k, m)-hyper-core is shown as a function of k at fixed values of m. All results are obtained by averaging the results of 10^3 numerical simulations, with a single random seed of infection and with an observation window $T = 10^3$. The (λ, ν) values considered for each data set are reported in Supplementary Table II and in all panels $\mu = 0.1$.

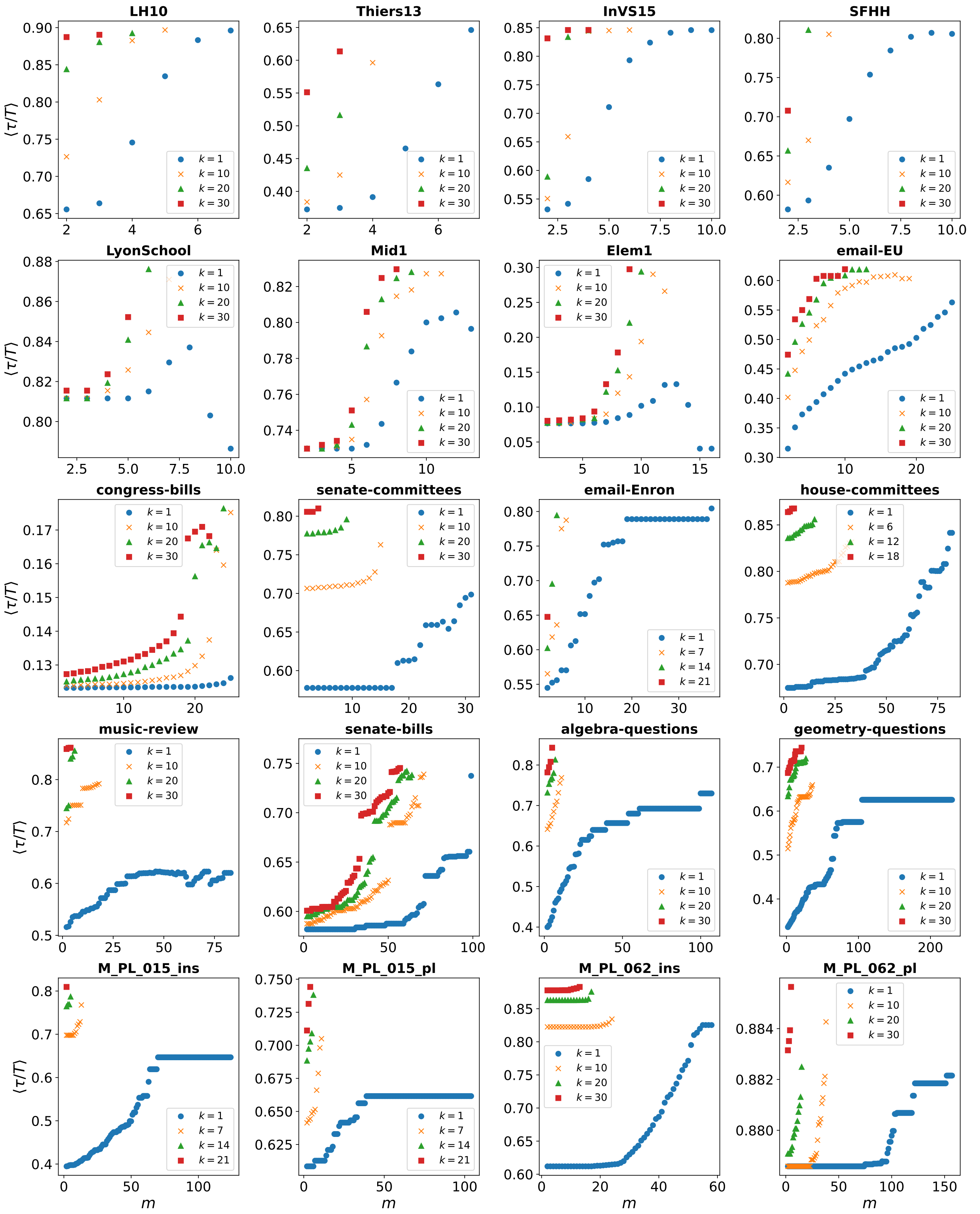

Supplementary Figure 18: **Higher-order non-linear contagion process - SIS model - III.** In all panels the average fraction $\langle \tau/T \rangle$ of time being infected in the SIS steady state averaged over the nodes of the (k, m)-hyper-core is shown as a function of m at fixed values of k. All results are obtained by averaging the results of 10^3 numerical simulations, with a single random seed of infection and with an observation window $T = 10^3$. The (λ, ν) values considered for each data set are reported in Supplementary Table II and in all panels $\mu = 0.1$.

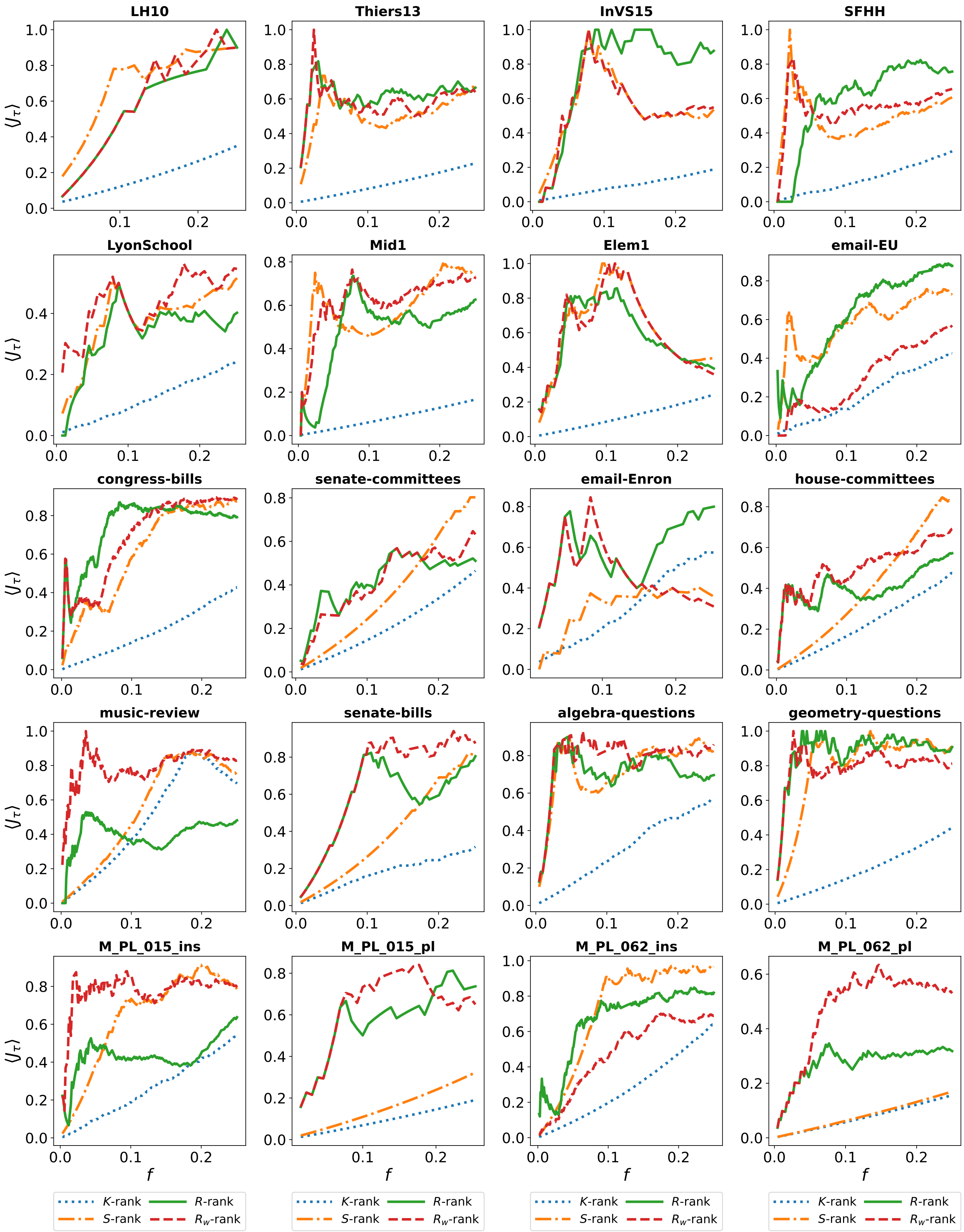

Supplementary Figure 19: **Higher-order non-linear contagion process - SIS model - IV.** All panels give, as a function of f, the average Jaccard similarity $\langle J_\tau \rangle$ between the nodes in the top fN positions of the rankings obtained through the dynamical property τ, i.e. time being infected in the SIS steady state, and each of the centralities considered. When some nodes has the same rank the similarity is averaged on all the possible combinations. All results are obtained by averaging the results of 10^3 numerical simulations, with a single random seed of infection and with an observation window $T = 10^3$. The (λ, ν) values considered for each data set are reported in Supplementary Table II and in all panels $\mu = 0.1$.

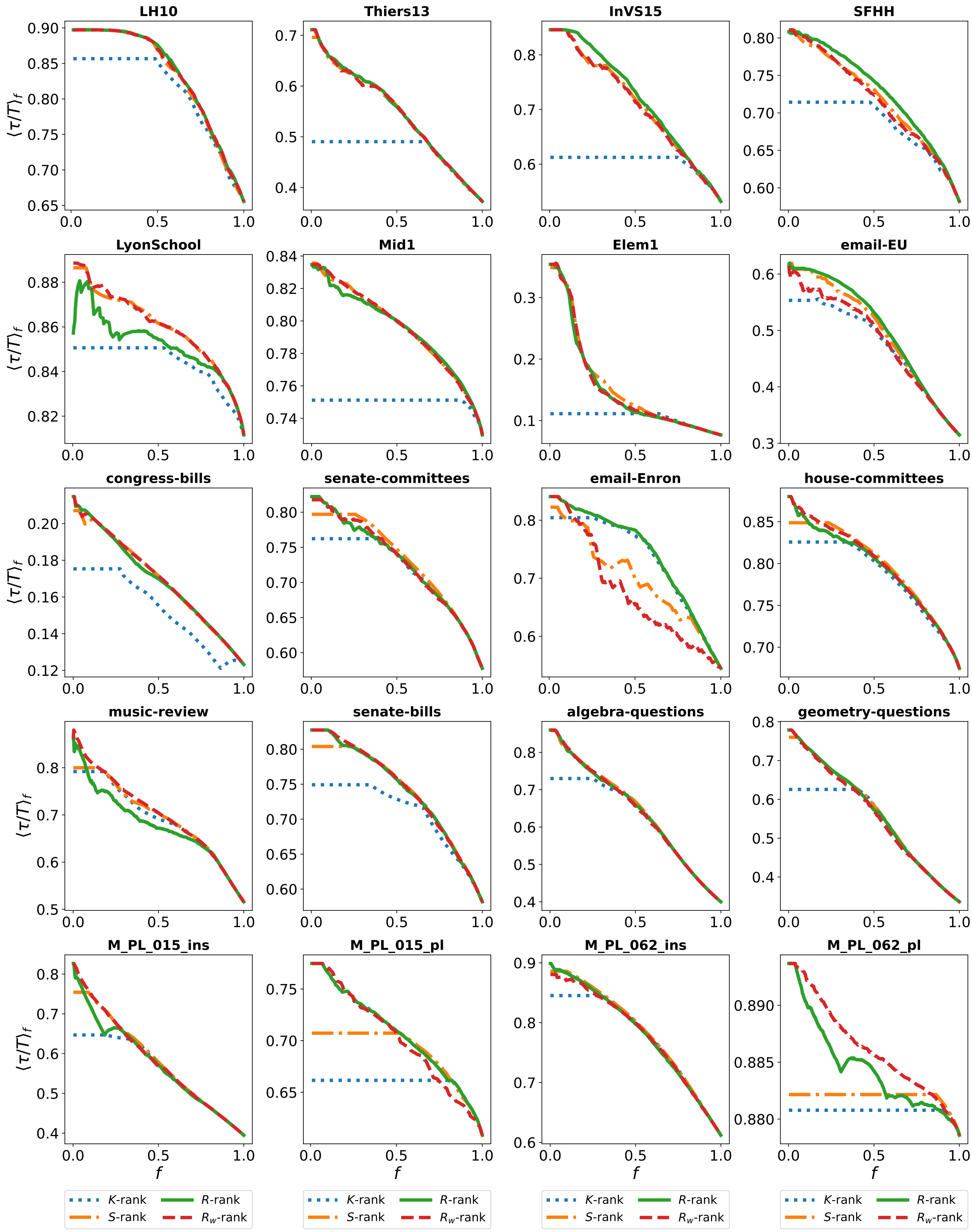

Supplementary Figure 20: **Higher-order non-linear contagion process - SIS model - V.** All panels give, the average fraction $\langle \tau/T \rangle_f$ of time being infected in the SIS steady state averaged over the first fN nodes according to the coreness rankings, as a function of f. All results are obtained by averaging the results of 10^3 numerical simulations, with a single random seed of infection and with an observation window $T = 10^3$. The (λ, ν) values considered for each data set are reported in Supplementary Table II and in all panels $\mu = 0.1$.

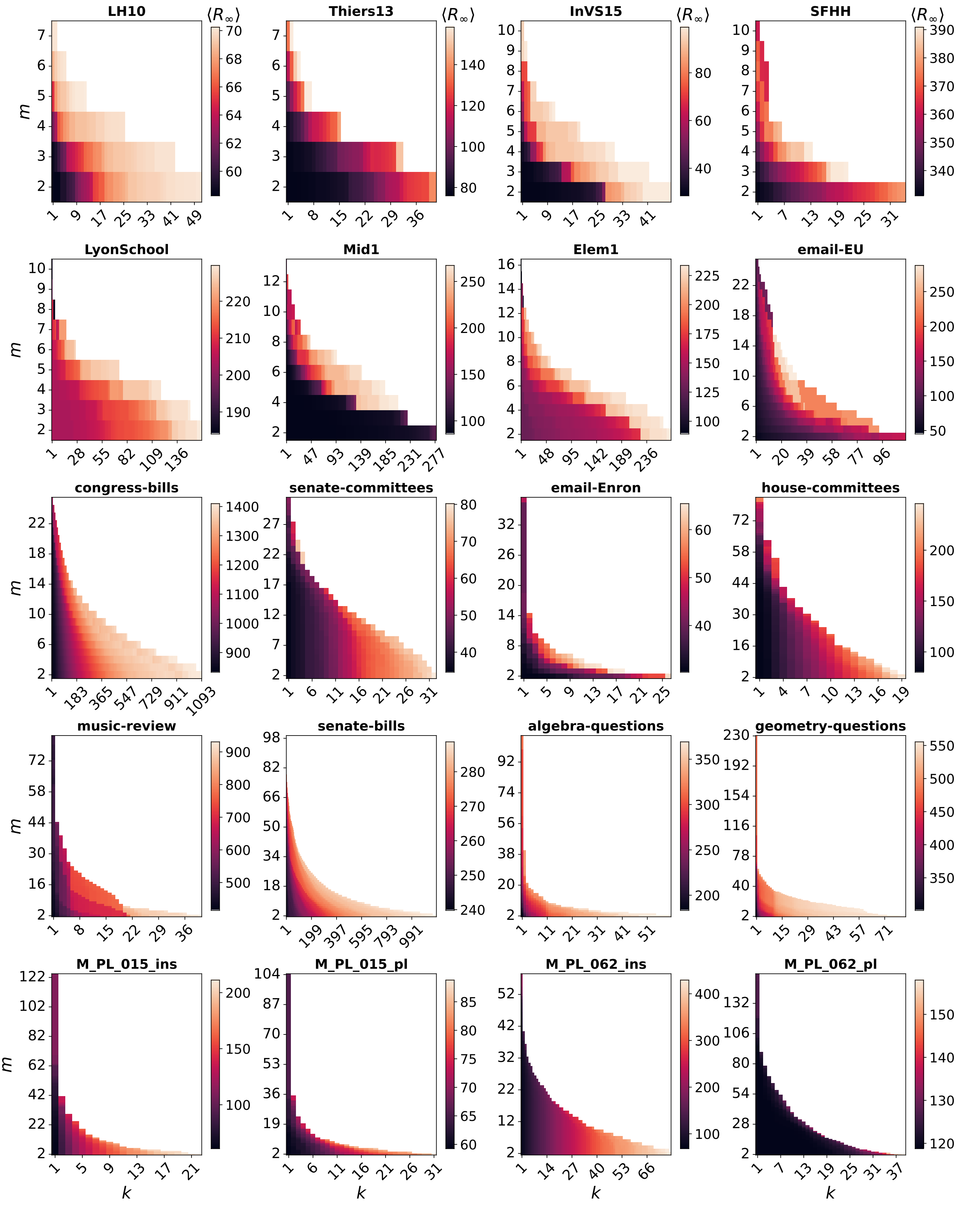

Supplementary Figure 21: **Higher-order non-linear contagion process - SIR model - I.** All panels show, as a function of k and m through a heat-map, the average epidemic final-size $\langle R_\infty \rangle$ produced by seeding the SIR process in a single seed belonging to the (k, m)-hyper-core (averaged over all nodes of the hyper-core). All results are obtained by averaging the results of 300 numerical simulations for each seed (except for the congress-bills data set which is the result of 10 simulations). The (λ, ν) values considered for each data set are reported in Supplementary Table III and in all panels $\mu = 0.1$.

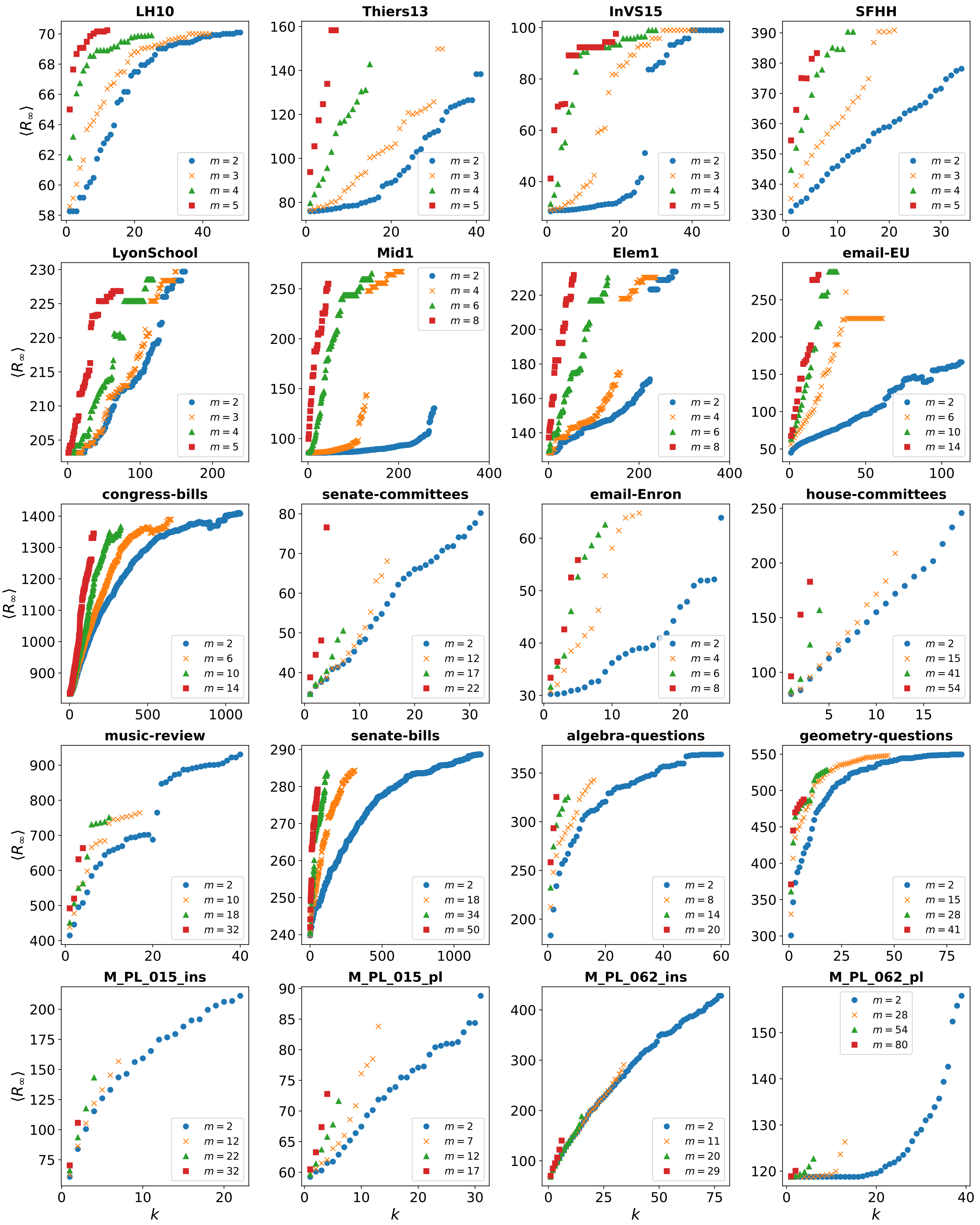

Supplementary Figure 22: **Higher-order non-linear contagion process - SIR model - II.** In all panels the average epidemic final-size $\langle R_\infty \rangle$ produced by seeding the SIR process in a single seed belonging to the (k, m)-hyper-core (averaged over all nodes of the hyper-core) is shown as a function of k at fixed values of m. All results are obtained by averaging the results of 300 numerical simulations for each seed (except for the congress-bills data set which is the result of 10 simulations). The (λ, ν) values considered for each data set are reported in Supplementary Table III and in all panels $\mu = 0.1$.

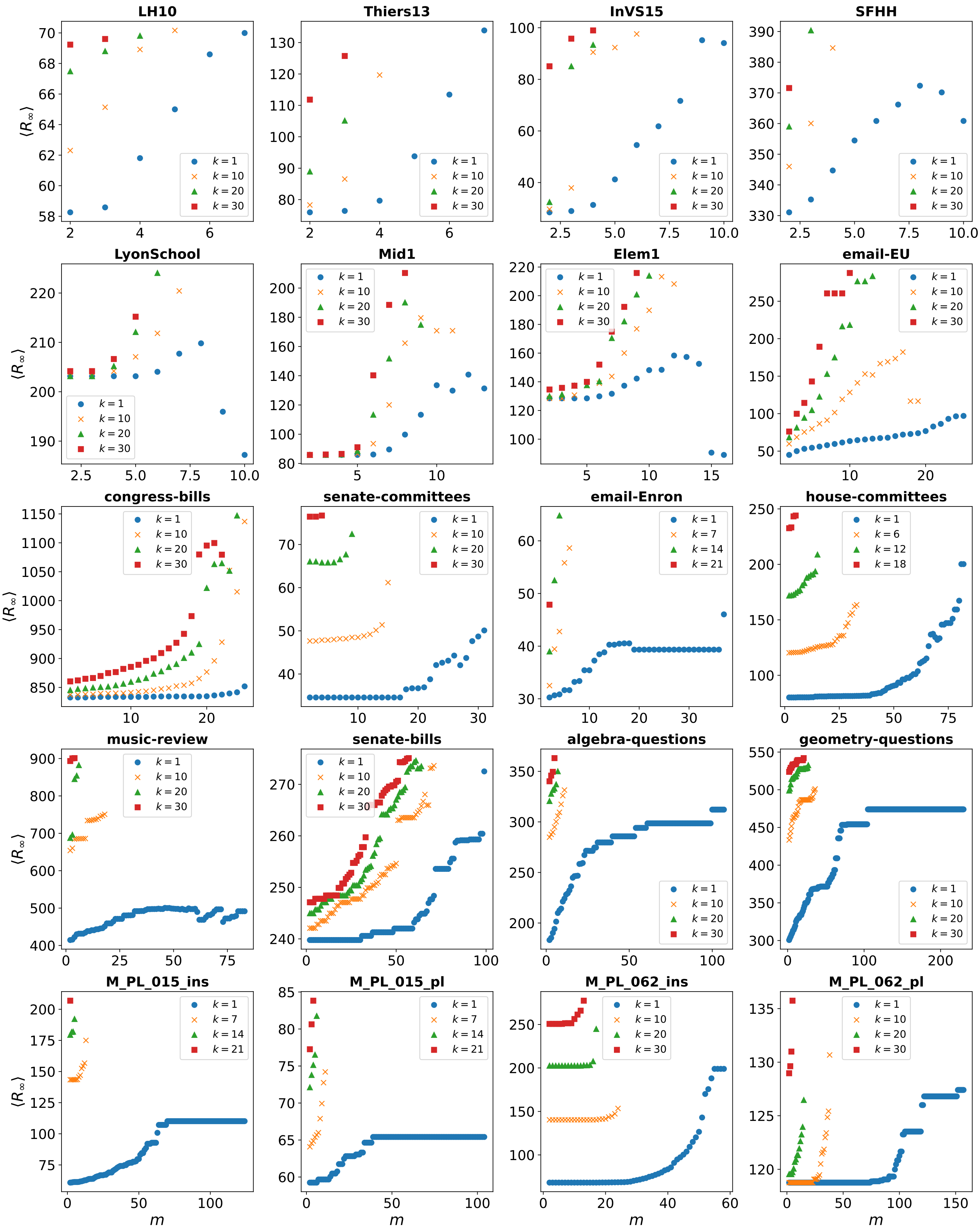

Supplementary Figure 23: **Higher-order non-linear contagion process - SIR model - III.** In all panels the average epidemic final-size $\langle R_\infty \rangle$ produced by seeding the SIR process in a single seed belonging to the (k, m)-hyper-core (averaged over all nodes of the hyper-core) is shown as a function of m at fixed values of k. All results are obtained by averaging the results of 300 numerical simulations for each seed (except for the congress-bills data set which is the result of 10 simulations). The (λ, ν) values considered for each data set are reported in Supplementary Table III and in all panels $\mu = 0.1$.

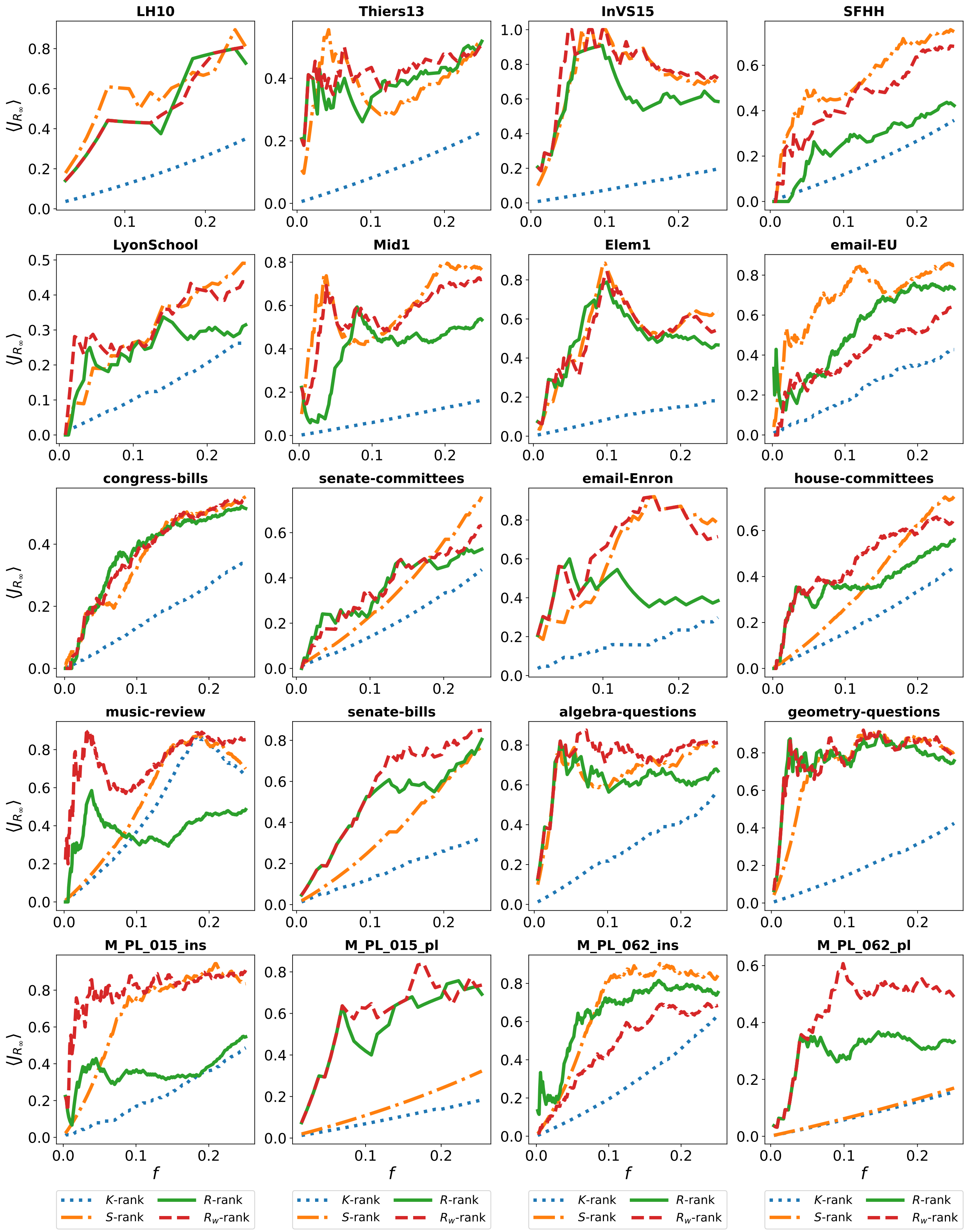

Supplementary Figure 24: **Higher-order non-linear contagion process - SIR model - IV.** All panels show, as a function of f, the average Jaccard similarity $\langle J_{R_\infty} \rangle$ between the nodes in the top fN positions of the rankings obtained through the dynamical property R_∞, i.e. the average epidemic final-size produced by seeding the SIR process in a single seed, and each of the centralities considered. When some nodes has the same rank the similarity is averaged on all the possible combinations. All results are obtained by averaging the results of 300 numerical simulations for each seed (except for the congress-bills data set which is the result of 10 simulations). The (λ, ν) values considered for each data set are reported in Supplementary Table III and in all panels $\mu = 0.1$.

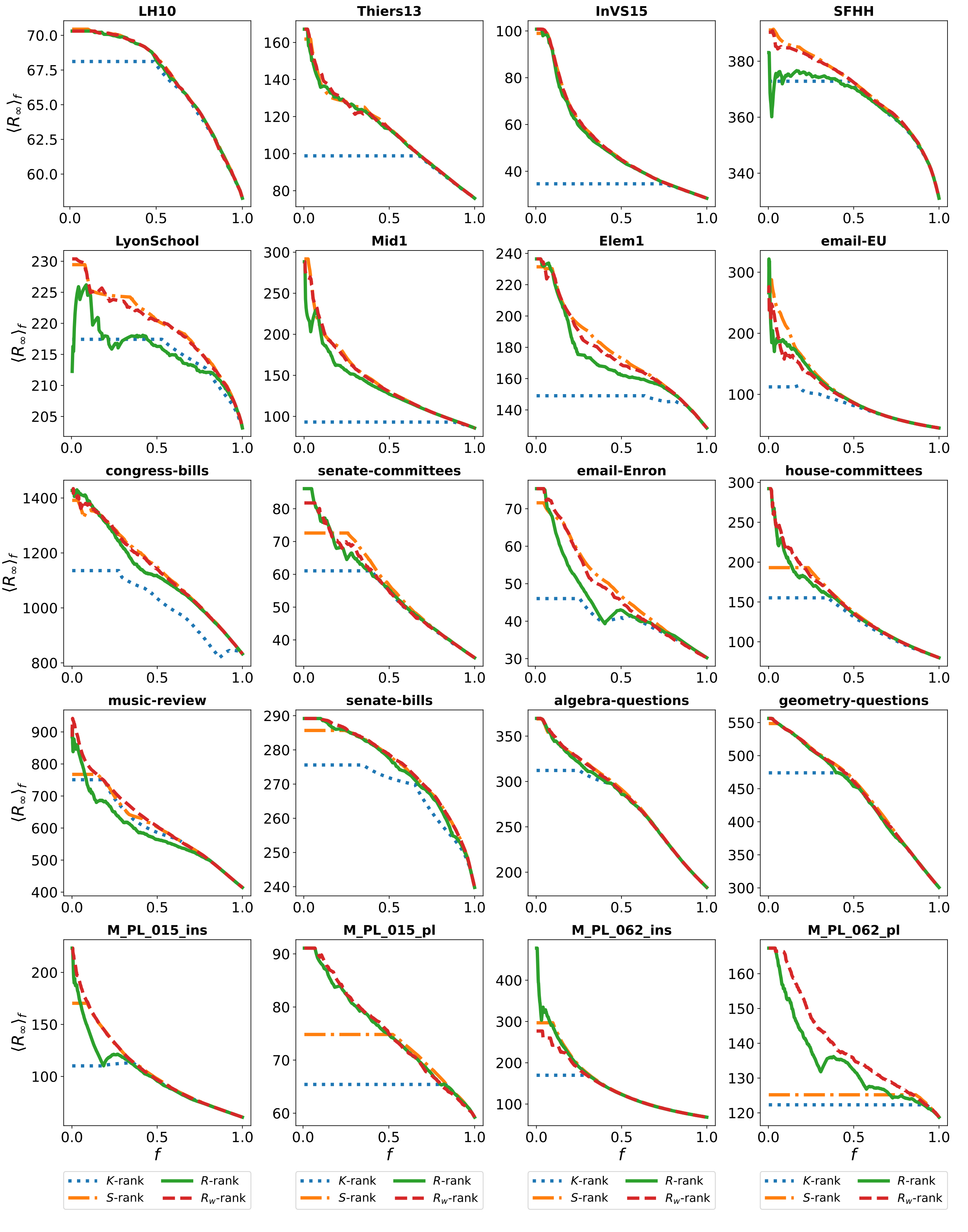

Supplementary Figure 25: **Higher-order non-linear contagion process - SIR model - V.** All panels show the average epidemic final-size $\langle R_\infty \rangle_f$ produced by seeding the SIR process in a single seed, averaged over the first fN nodes according to coreness rankings, as a function of f. All results are obtained by averaging the results of 300 numerical simulations for each seed (except for the congress-bills data set which is the result of 10 simulations). The (λ, ν) values considered for each data set are reported in Supplementary Table III and in all panels $\mu = 0.1$.

V. SUPPLEMENTARY NOTE 5: THRESHOLD HIGHER-ORDER CONTAGION PROCESS

In this Supplementary Note we consider another spreading process in which multi-body interactions drive the infection through a threshold effect and group contagion [4, 6]: the threshold higher-order contagion process. We consider both the SIR and SIS epidemic models on static hypergraphs: for each hyperedge of size m in which i individuals are in the state I, if the fraction of infected individuals i/m is larger or equal to a threshold θ, i.e. if $i \geq \lceil \theta m \rceil$, a group infection is activated at rate λ and the susceptible nodes in the hyperedge become all infected. Note that if we consider a single seed of infection: for $\theta \leq 1/M$ the group infection is activated in all the hyperedges containing the seed; for $\theta = 1/m$ the spreading is activated only in the hyperedges containing the seed that have size smaller or equal to m; for $\theta > 1/2$ the spreading is inhibited since more than one infected node is required to activate the infection in all hyperedges. I individuals recover independently at constant rate μ, becoming either S (SIS model) or R (SIR model).

We perform numerical simulations of this process, for both SIS and SIR models, on empirical hypergraphs: the simulation procedures are analogous to those described in the main text for the higher-order non-linear contagion process (see Methods), since the two processes only differ in the infection mechanism. In the threshold higher-order contagion, for each time-step Δt, given a hyperedge of size m containing i infected nodes, if $i \geq \lceil \theta m \rceil$ a group infection process is activated with probability λ and all susceptible nodes in the hyperedge are infected. Thus, in each time-step each of the interaction groups respecting the condition $i \geq \lceil \theta m \rceil$ produces a group infection process with probability λ.

Therefore, also in this case we quantify the "spreading power" of each node considered separately as seed for the SIR model and the nodes on which the epidemic is mainly localized in the steady state, i.e. the nodes that drive and sustain the process, for the SIS model. In Supplementary Figs. 26-30 we show the results of SIS simulations and in Supplementary Figs. 31-35 the results of SIR simulations, also comparing the performance of different coreness centralities in identifying central nodes for the dynamic processes (Supplementary Figs. 29-30 and Supplementary Figs. 34-35).

**data set**	θ	λ
LH10	0.03	0.005
Thiers13	1/7	0.001
InVS15	1/10	0.001
SFHH	1/10	0.001
LyonSchool	0.15	0.001
Mid1	0.03	0.001
Elem1	0.03	0.001
email-EU	0.03	0.001
congress-bills	0.03	0.001
senate-committees	0.03	0.01

**data set**	θ	λ
email-Enron	1/37	0.01
house-committees	0.03	0.01
music-review	0.03	0.01
senate-bills	1/99	0.0001
algebra-questions	1/107	0.001
geometry-questions	0.03	0.001
M_PL_015_ins	1/124	0.005
M_PL_015_pl	1/104	0.01
M_PL_062_ins	1/58	0.005
M_PL_062_pl	1/157	0.005

Supplementary Table IV: **Parameters for Supplementary Figs. 26-30.** The tables summarize the parameters of the threshold higher-order SIS contagion process considered for each data set in Supplementary Figs. 26-30.

**data set**	θ	λ
LH10	0.03	0.01
Thiers13	1/7	0.001
InVS15	1/10	0.01
SFHH	0.03	0.01
LyonSchool	0.15	0.01
Mid1	1/13	0.001
Elem1	0.03	0.001
email-EU	0.03	0.001
congress-bills	0.03	0.001
senate-committees	0.15	0.01

**data set**	θ	λ
email-Enron	0.3	0.01
house-committees	1/82	0.01
music-review	1/83	0.01
senate-bills	0.3	0.001
algebra-questions	1/107	0.001
geometry-questions	1/230	0.001
M_PL_015_ins	1/124	0.005
M_PL_015_pl	1/104	0.005
M_PL_062_ins	1/58	0.005
M_PL_062_pl	1/157	0.005

Supplementary Table V: **Parameters for Supplementary Figs. 31-35.** The tables summarize the parameters of the threshold higher-order SIR contagion process considered for each data set in Supplementary Figs. 31-35.

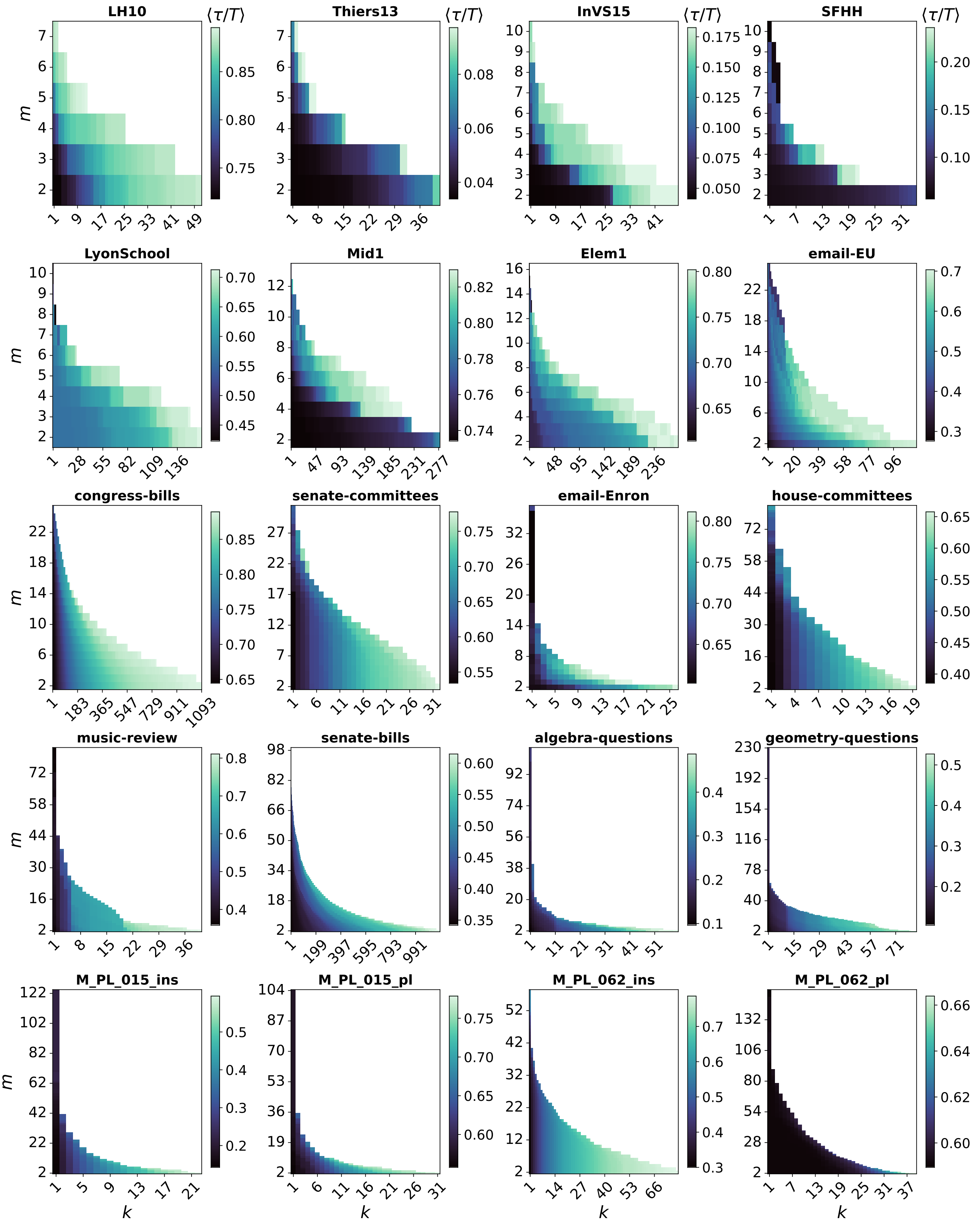

Supplementary Figure 26: **Threshold higher-order contagion process - SIS model - I.** All panels give, as a heat-map as a function of k and m, the average fraction $\langle \tau/T \rangle$ of time being infected in the SIS steady state averaged over the nodes of the (k, m)-hyper-core. All results are obtained by averaging the results of 10^3 numerical simulations, with a single random seed of infection and with an observation window $T = 10^3$. The (λ,θ) values considered for each data set are summarized in Supplementary Table IV and in all panels $\mu = 0.1$.

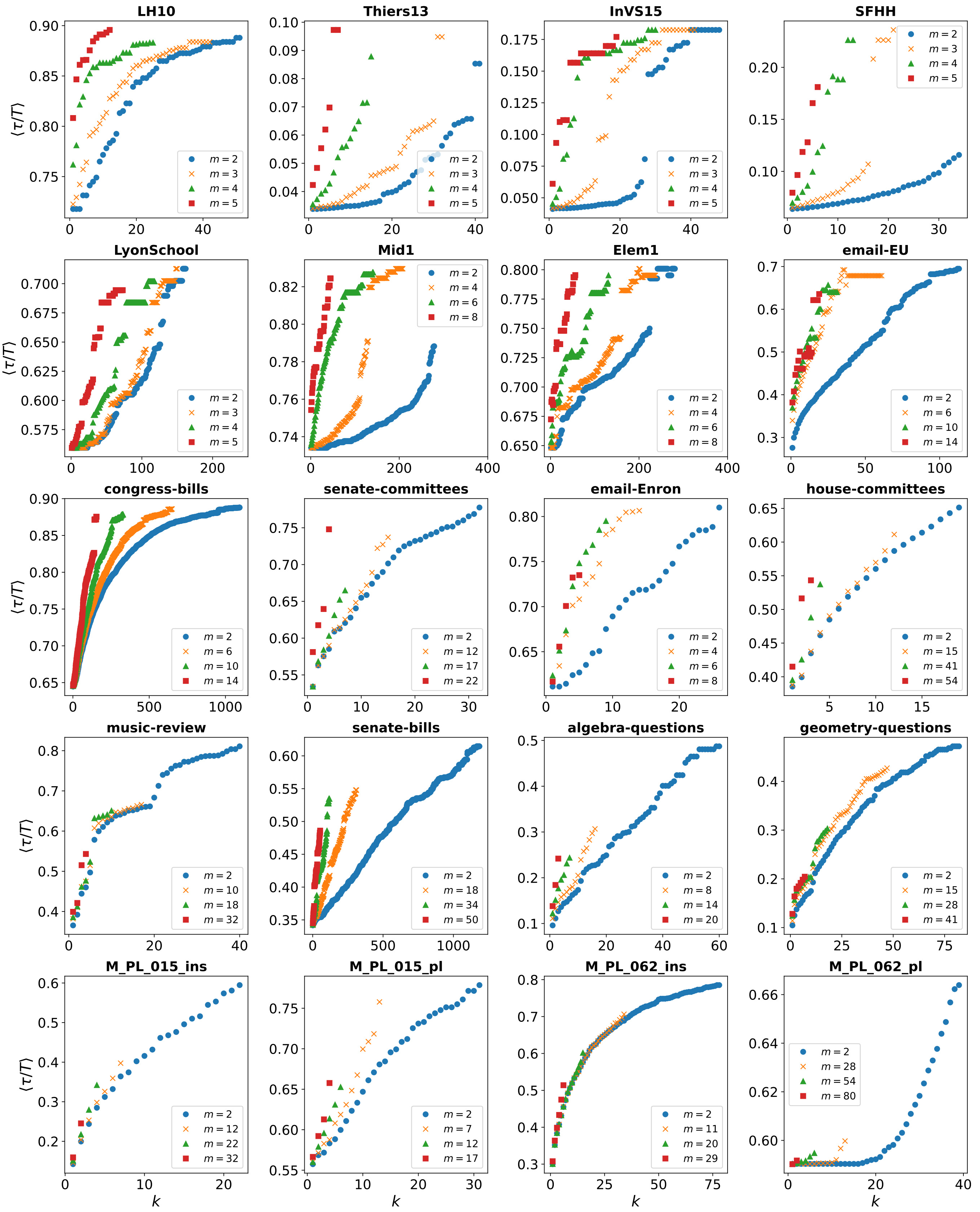

Supplementary Figure 27: **Threshold higher-order contagion process - SIS model - II.** In all panels the average fraction $\langle \tau/T \rangle$ of time being infected in the steady state averaged over the nodes of the (k,m)-hyper-core is shown as a function of k at fixed values of m. All results are obtained by averaging the results of 10^3 numerical simulations, with a single random seed of infection and with an observation window $T = 10^3$. The (λ,θ) values considered for each data set are summarized in Supplementary Table IV and in all panels $\mu = 0.1$.

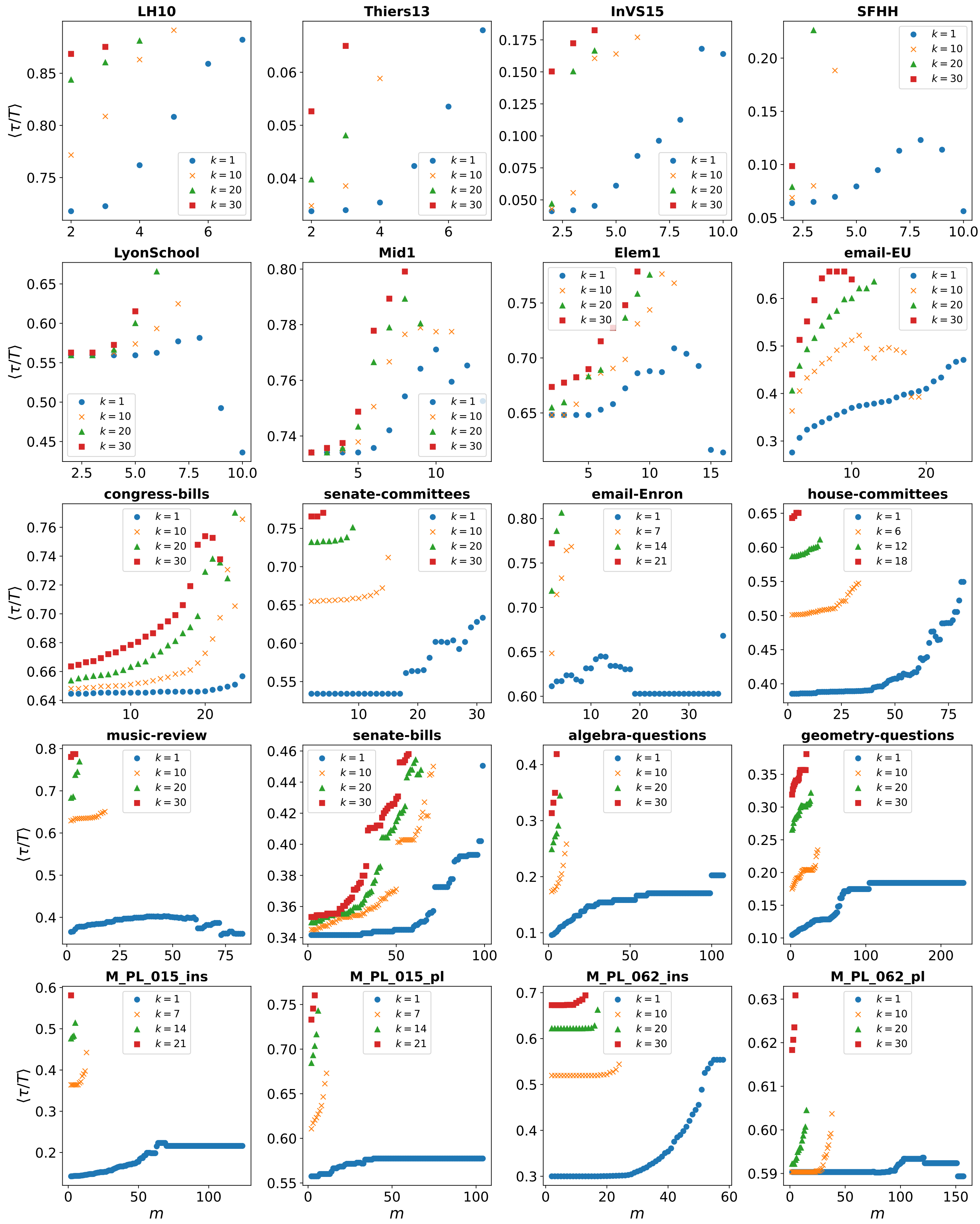

Supplementary Figure 28: **Threshold higher-order contagion process - SIS model - III.** In all panels the average fraction $\langle\tau/T\rangle$ of time being infected in the steady state averaged over the nodes of the (k,m)-hyper-core is shown as a function of m at fixed values of k. All results are obtained by averaging the results of 10^3 numerical simulations, with a single random seed of infection and with an observation window $T = 10^3$. The (λ,θ) values considered for each data set are summarized in Supplementary Table IV and in all panels $\mu = 0.1$.

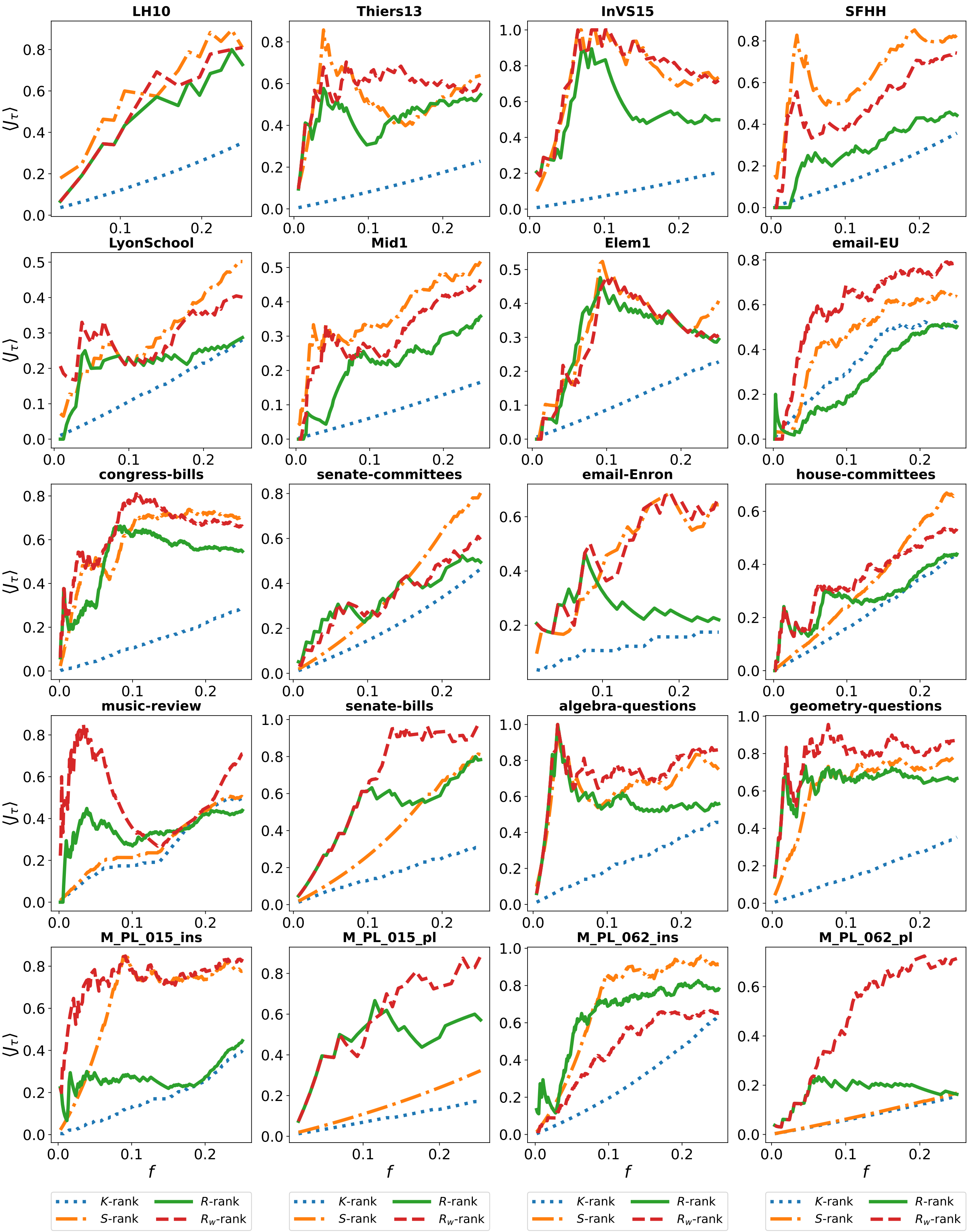

Supplementary Figure 29: **Threshold higher-order contagion process - SIS model - IV.** All panels give, as a function of f, the average Jaccard similarity $\langle J_\tau \rangle$ between the nodes in the top fN positions of the rankings obtained through the dynamical property τ, i.e. time being infected in the SIS steady state, and each of the centralities considered. When some nodes has the same rank the similarity is averaged on all the possible combinations. All results are obtained by averaging the results of 10^3 numerical simulations, with a single random seed of infection and with an observation window $T = 10^3$. The (λ,θ) values considered for each data set are summarized in Supplementary Table IV and in all panels $\mu = 0.1$.

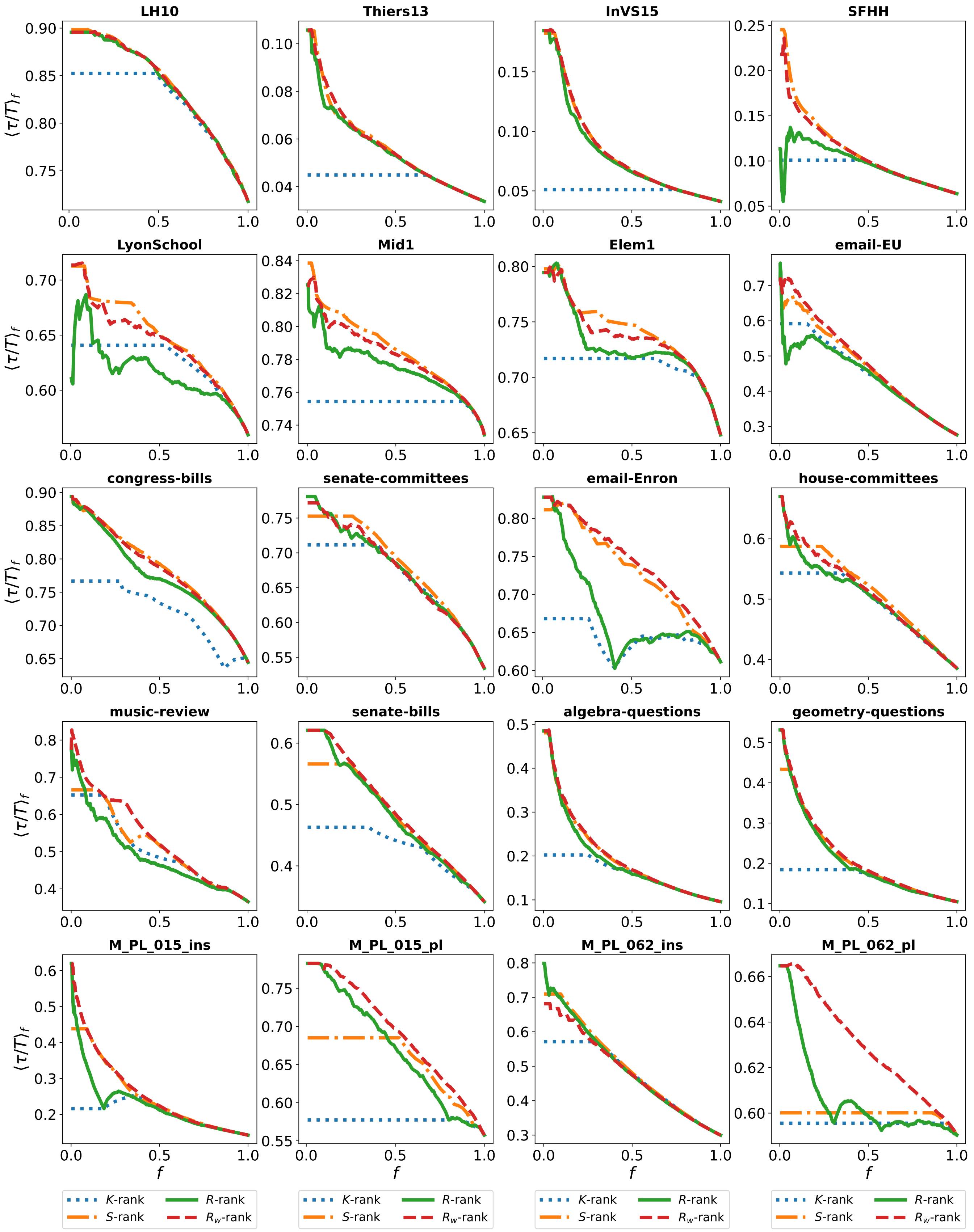

Supplementary Figure 30: **Threshold higher-order contagion process - SIS model - V.** All panels give, the average fraction $\langle\tau/T\rangle_f$ of time being infected in the SIS steady state averaged over the first fN nodes according to the coreness rankings, as a function of f. All results are obtained by averaging the results of 10^3 numerical simulations, with a single random seed of infection and with an observation window $T = 10^3$. The (λ,θ) values considered for each data set are summarized in Supplementary Table IV and in all panels $\mu = 0.1$.

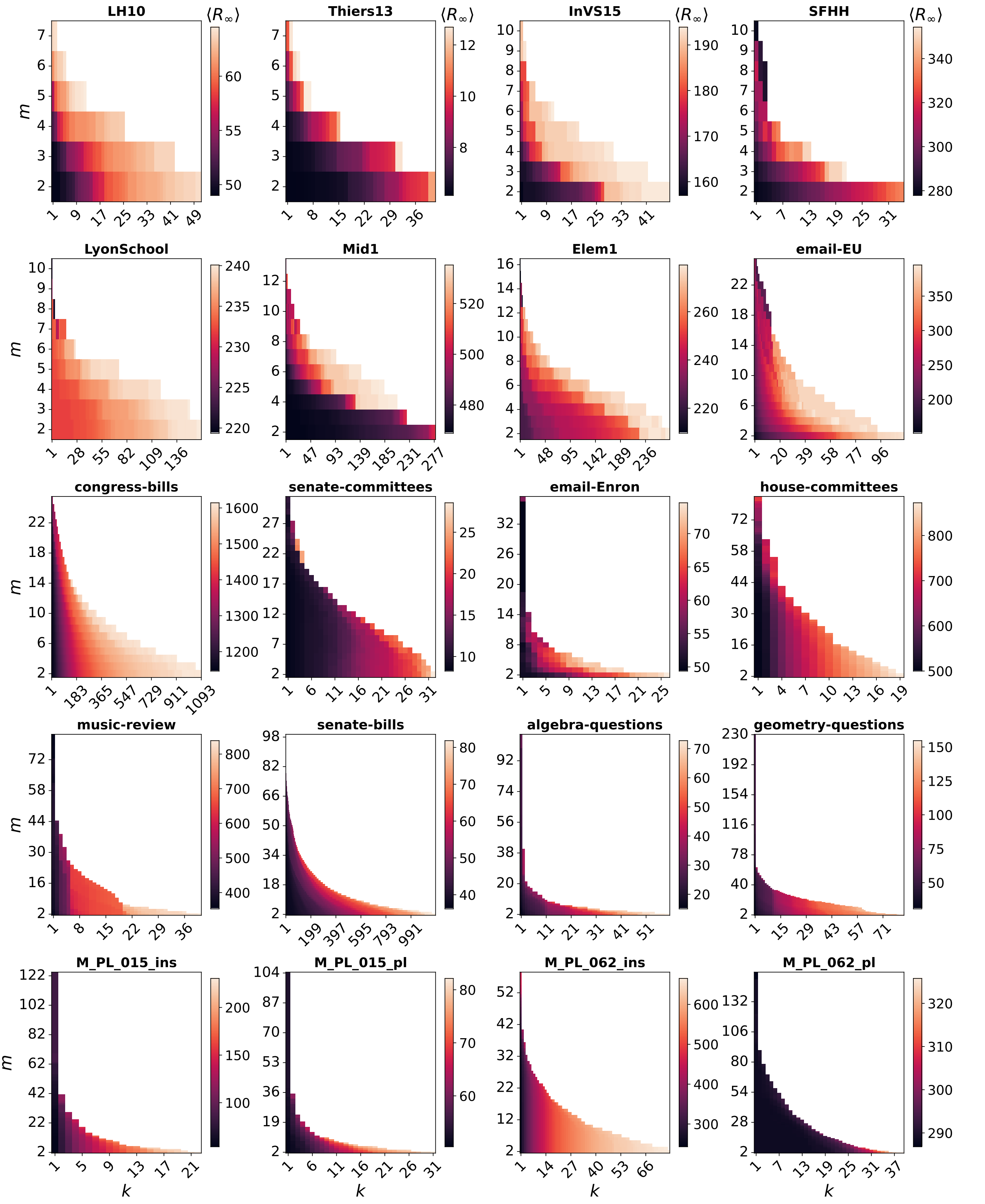

Supplementary Figure 31: **Threshold higher-order contagion process - SIR model - I.** All panels show, as a function of k and m through a heat-map, the average epidemic final-size $\langle R_\infty \rangle$ produced by seeding the SIR process in a single seed belonging to the (k, m)-hyper-core (averaged over all nodes of the hyper-core). All results are obtained by averaging the results of 300 numerical simulations for each seed (except for the congress-bills data set which is the result of 10 simulations). The (λ,θ) values considered for each data set are summarized in Supplementary Table V and in all panels $\mu = 0.1$.

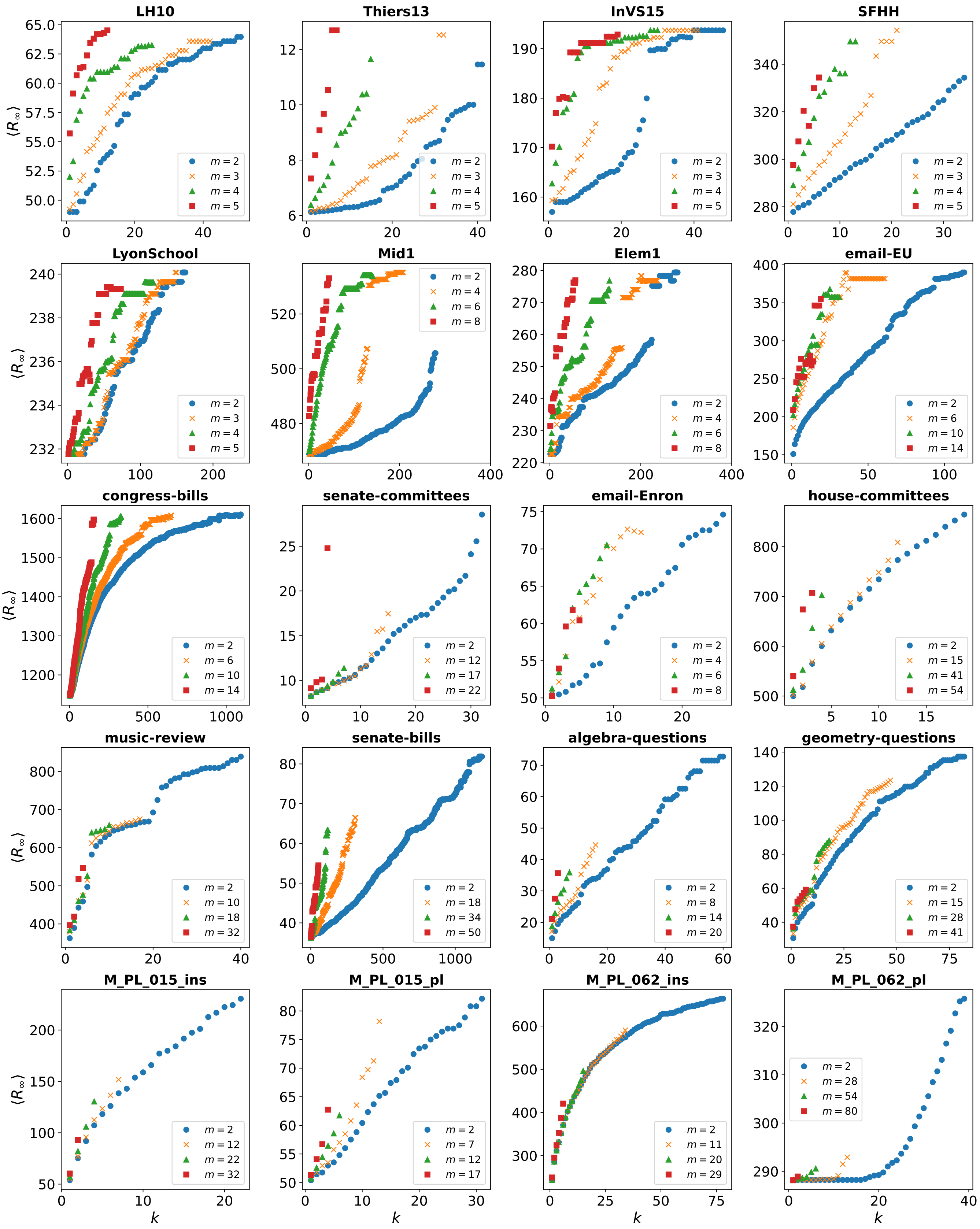

Supplementary Figure 32: **Threshold higher-order contagion process - SIR model - II.** In all panels the average epidemic final-size $\langle R_\infty \rangle$ produced by seeding the SIR process in a single seed belonging to the (k, m)-hyper-core (averaged over all nodes of the hyper-core) is shown as a function of k at fixed values of m. All results are obtained by averaging the results of 300 numerical simulations for each seed (except for the congress-bills data set which is the result of 10 simulations). The (λ,θ) values considered for each data set are summarized in Supplementary Table V and in all panels $\mu = 0.1$.

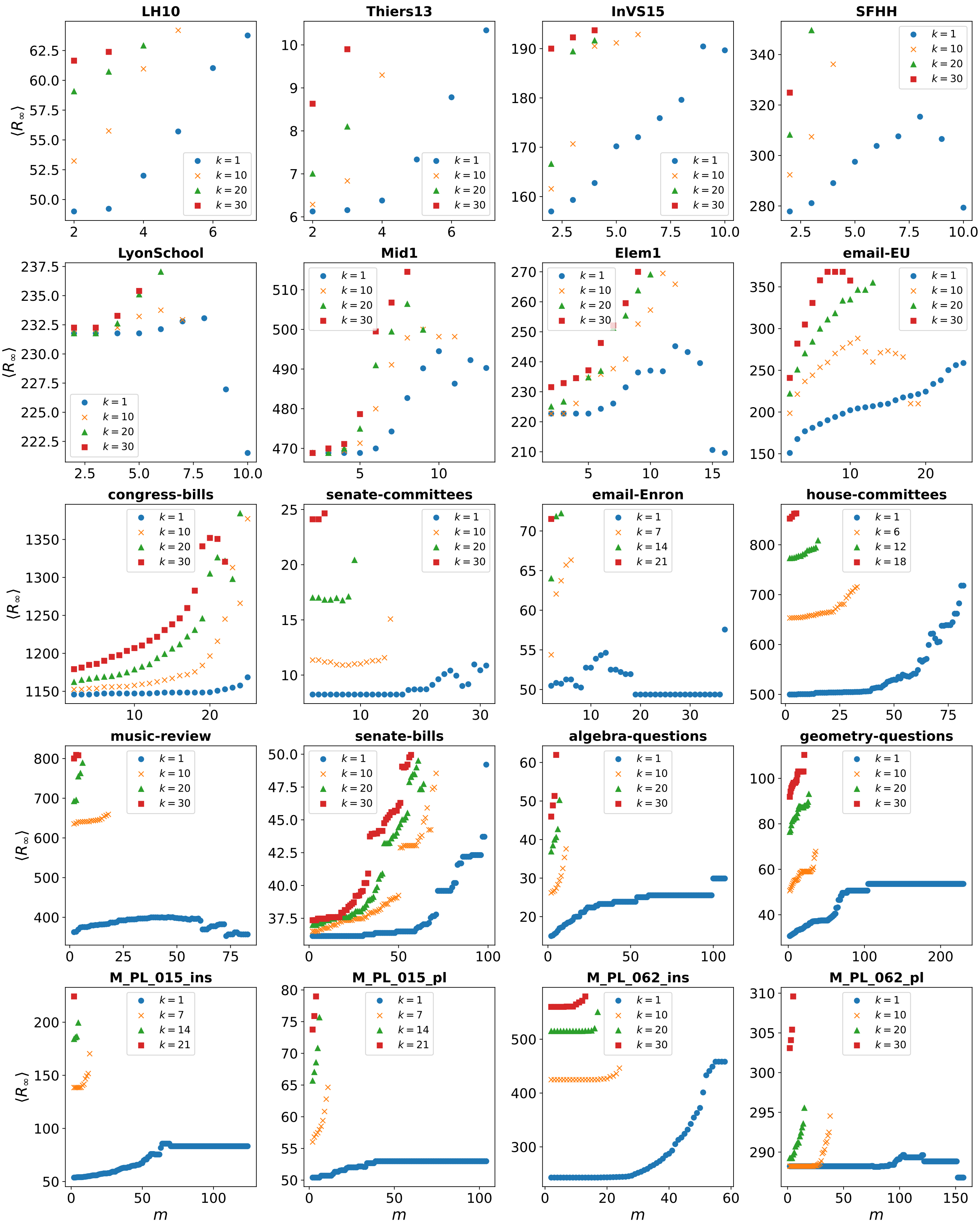

Supplementary Figure 33: **Threshold higher-order contagion process - SIR model - III.** In all panels the average epidemic final-size $\langle R_\infty \rangle$ produced by seeding the SIR process in a single seed belonging to the (k, m)-hyper-core (averaged over all nodes of the hyper-core) is shown as a function of m at fixed values of k. All results are obtained by averaging the results of 300 numerical simulations for each seed (except for the congress-bills data set which is the result of 10 simulations). The (λ,θ) values considered for each data set are summarized in Supplementary Table V and in all panels $\mu = 0.1$.

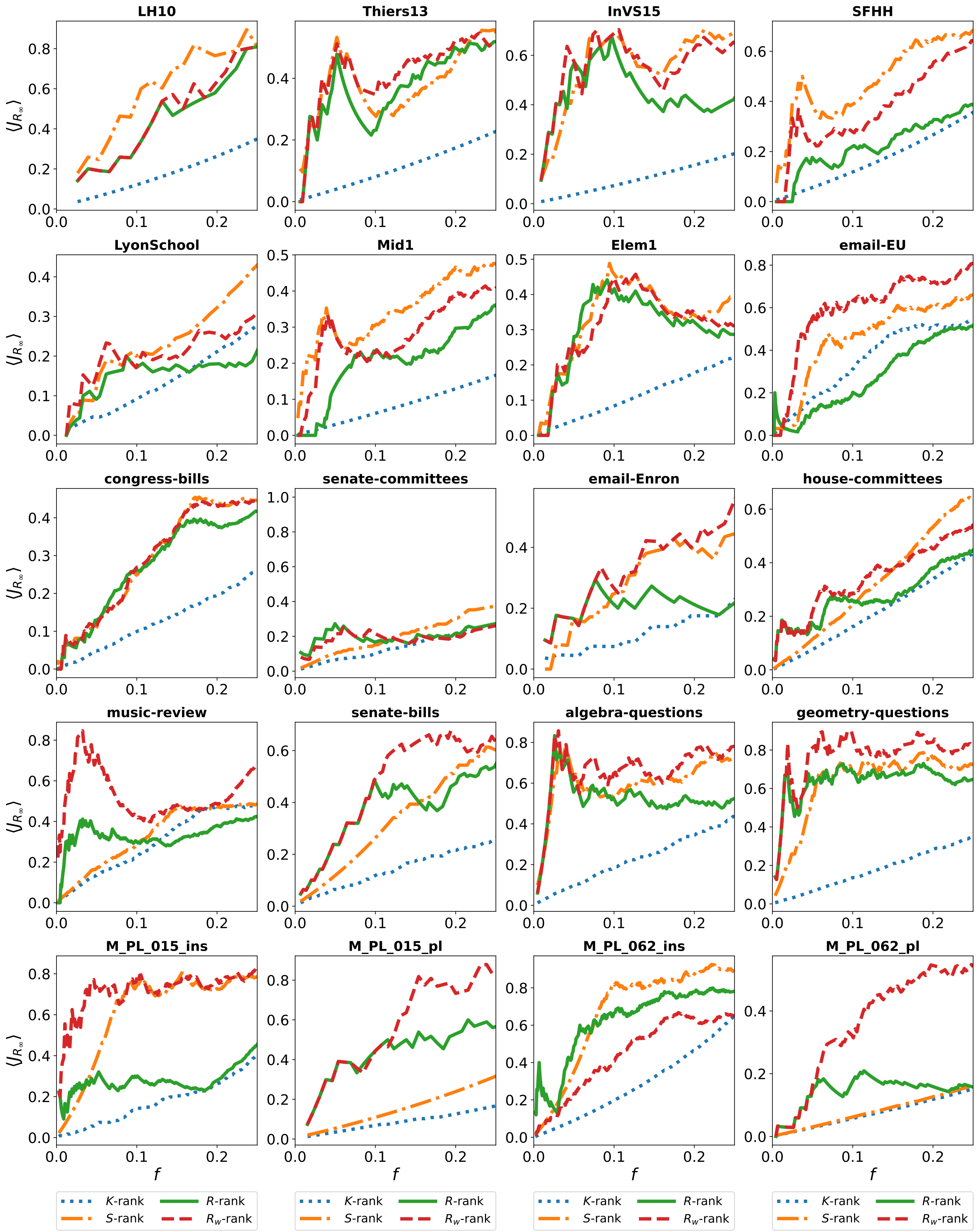

Supplementary Figure 34: **Threshold higher-order contagion process - SIR model - IV.** All panels show, as a function of f, the average Jaccard similarity $\langle J_{R_\infty} \rangle$ between the nodes in the top fN positions of the rankings obtained through the dynamical property R_∞, i.e. the epidemic final size produced by seeding the SIR process in a single seed, and each of the centralities considered. When some nodes has the same rank the similarity is averaged on all the possible combinations. All results are obtained by averaging the results of 300 numerical simulations for each seed (except for the congress-bills data set which is the result of 10 simulations). The (λ,θ) values considered for each data set are summarized in Supplementary Table V and in all panels $\mu = 0.1$.

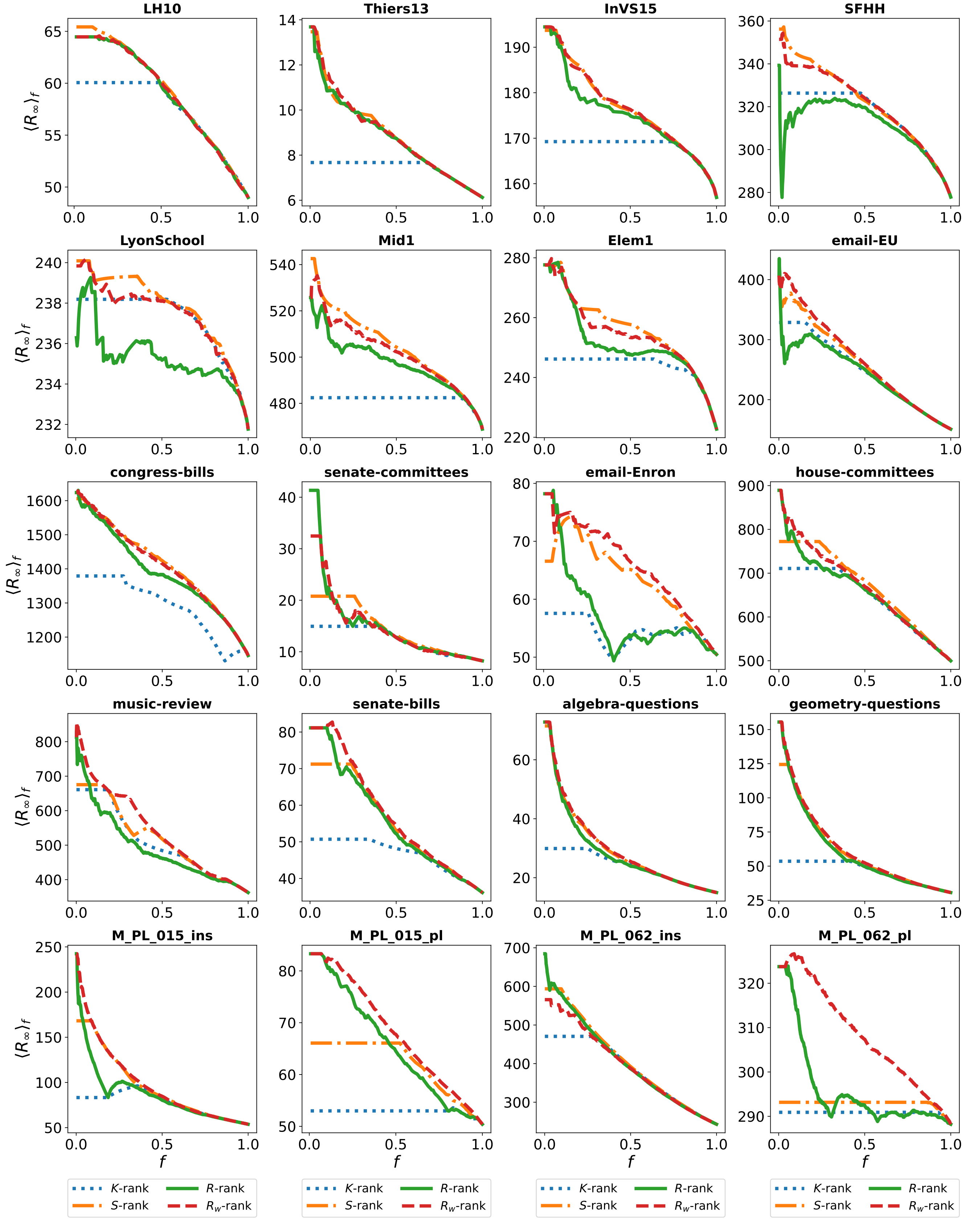

Supplementary Figure 35: **Threshold higher-order contagion process - SIR model - V.** All panels show the average epidemic final-size $\langle R_\infty \rangle_f$ produced by seeding the SIR process in a single seed, averaged over the first fN nodes according to coreness rankings, as a function of f. All results are obtained by averaging the results of 300 numerical simulations for each seed (except for the congress-bills data set which is the result of 10 simulations). The (λ,θ) values considered for each data set are summarized in Supplementary Table V and in all panels $\mu = 0.1$.

VI. SUPPLEMENTARY NOTE 6: HIGHER-ORDER NAMING-GAME (NG) PROCESS

In this Supplementary Note, we present the results of the higher-order naming-game process [5] both for the union (Supplementary Fig. 36,38) and the unanimity rules (Supplementary Fig. 37,39).

**data set**	β	p	t_{max}	T
InVS15	0.41	1.8×10^{-2}	10^5	10^4
Mid1	0.59	1.5×10^{-2}	10^5	10^4
email-EU	0.52	9.2×10^{-3}	5×10^5	5×10^4
congress-bills	0.59	2.4×10^{-2}	5×10^5	5×10^4
house-committees	0.45	7.8×10^{-3}	5×10^5	5×10^4
music-review	0.52	9.0×10^{-3}	10^5	10^4

Supplementary Table VI: **Parameters for Supplementary Figs. 36,38 - Union rule.** The table summarizes the main parameters of the higher-order naming-game process considered for the temporal dynamics of Supplementary Fig. 38 in the various data sets (union rule).

**data set**	**Random**	**k-core**	**s-core**	**hyper-core-R_w**	**hyper-core-R**
InVS15	24.3%	31.0%	54.8%	55.2%	54.8%
Mid1	14.5%	16.1%	25.5%	24.3%	23.7%
email-EU	37.0%	51.9%	45.9%	45.2%	56.4%
congress-bills	40.8%	44.3%	47.1%	48.3%	49.7%
house-committees	63.0%	63.6%	64.0%	64.8%	64.6%
music-review	51.6%	55.3%	55.8%	60.0%	59.5%

Supplementary Table VII: **Minority takeover areas for Supplementary Fig. 36 - Union rule.** The table reports the area A of the explored parameter space in which the minority take-over, i.e. $n_A^* = 1$, takes place for the different data sets of Supplementary Fig. 36 (union rule) and for the different strategies of committed seeding.

**data set**	β	p	t_{max}	T
InVS15	0.38	1.4×10^{-2}	10^5	10^4
Mid1	0.38	2.7×10^{-2}	10^5	10^4
email-EU	0.41	1.7×10^{-2}	5×10^5	5×10^4
congress-bills	0.48	2.3×10^{-2}	5×10^5	5×10^4
house-committees	0.41	3.1×10^{-3}	5×10^5	5×10^4
music-review	0.52	1.0×10^{-2}	10^5	10^4

Supplementary Table VIII: **Parameters for Supplementary Fig. 37,39 - Unanimity rule.** The table summarizes the main parameters of the higher-order naming-game process considered for the temporal dynamics of Supplementary Fig. 39 in the various data sets (unanimity rule).

**data set**	**Random**	**k-core**	**s-core**	**hyper-core-R_w**	**hyper-core-R**
InVS15	8.6%	11.0%	35.2%	34.3%	32.9%
Mid1	1.0%	1.2%	2.5%	2.5%	2.5%
email-EU	7.3%	38.0%	8.6%	29.4%	14.7%
congress-bills	7.9%	13.8%	16.3%	23.0%	41.5%
house-committees	54.6%	61.3%	63.3%	64.5%	64.4%
music-review	33.9%	55.8%	56.6%	64.5%	62.6%

Supplementary Table IX: **Minority takeover areas for Supplementary Fig. 37 - Unanimity rule.** The table reports the area A of the explored parameter space in which the minority take-over, i.e. $n_A^* = 1$, takes place for the different data sets of Supplementary Fig. 37 (unanimity rule) and for the different strategies of committed seeding.

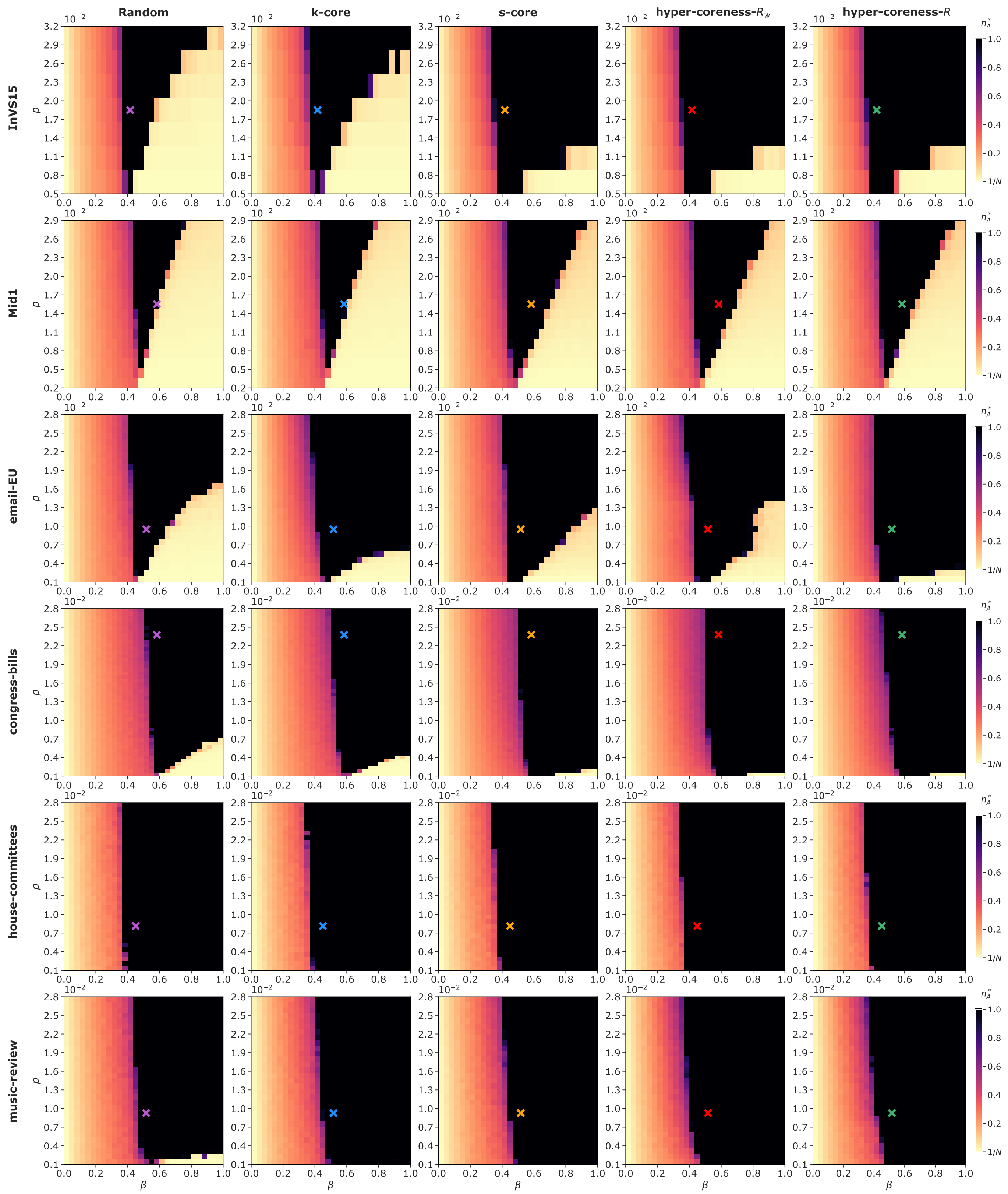

Supplementary Figure 36: **Higher-order NG process - Union rule.** The stationary fraction n_A^* of nodes supporting only the name A is shown as a function of the fraction of committed nodes p and the agreement probability β through a heat-map. We consider the union rule and the following data sets: InVS15 (first row), Mid1 (second row), email-EU (third row), congress-bills (fourth row), house-committees (fifth row), music-reviews (sixth row). For each row, the committed nodes are selected through: the random, the top k-coreness, the top s-coreness, the top frequency-based R_w-hyper-coreness and the top size-independent R-hyper-coreness seeding strategies. The minority take-over, i.e. $n_A^* = 1$, takes place over an area A of the explored parameter space: its value, for each strategy, is reported in Supplementary Table VII. All simulations are run until the absorbing state with $n_A^* = 1$ is reached or the dynamics has evolved for t_{max} time steps and the stationary fraction n_A^* is obtained by averaging over 100 values sampled in the last T time-steps (see Supplementary Table VI for the t_{max} and T values for each data set). The results refer to the median values obtained over 200 simulations. Cross markers in the heatmaps indicate the (β, p) values considered for Supplementary Fig. 38.

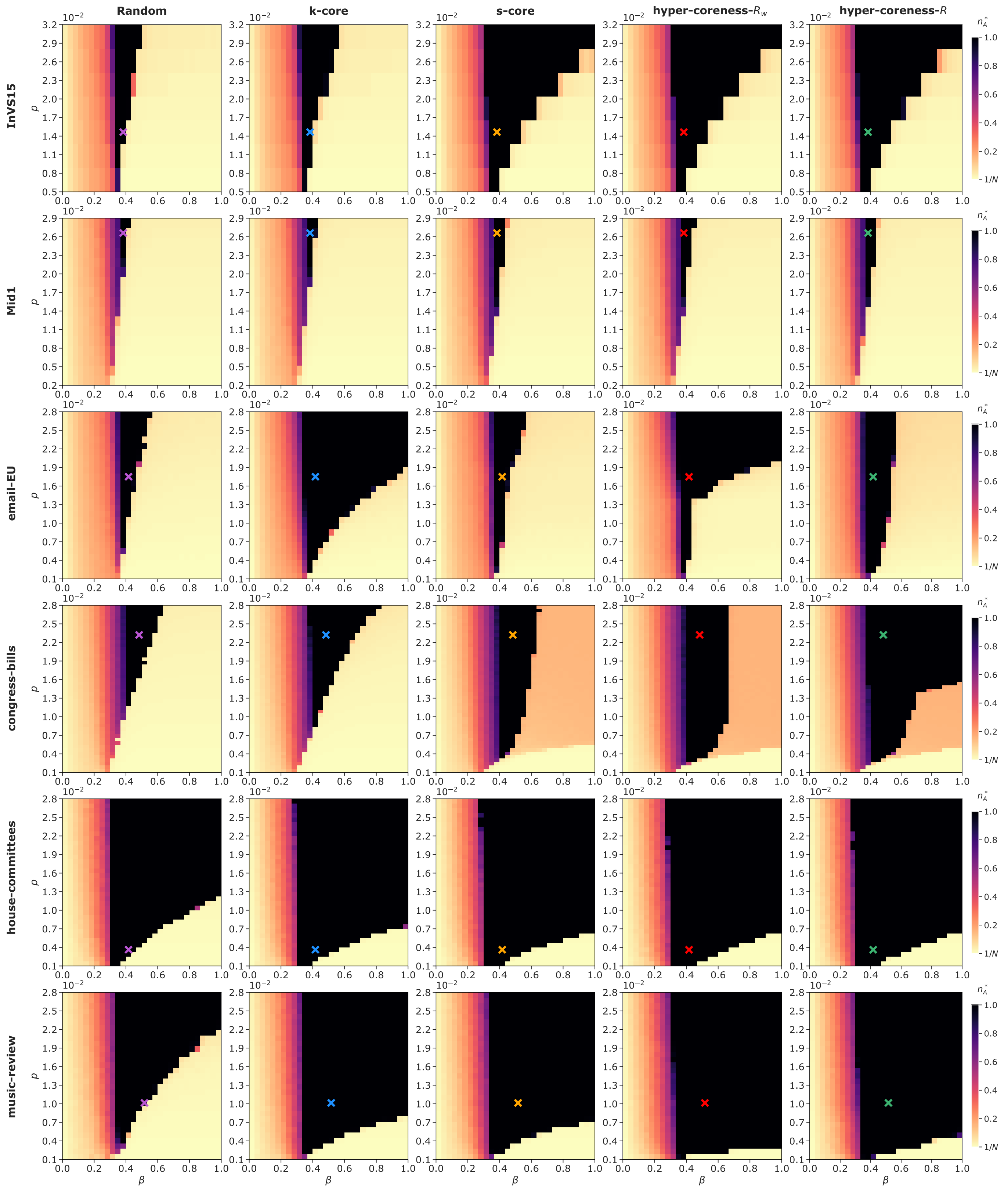

Supplementary Figure 37: **Higher-order NG process - Unanimity rule.** The stationary fraction n_A^* of nodes supporting only the name A is shown as a function of the fraction of committed nodes p and the agreement probability β through a heat-map. We consider the unanimity rule and the following data sets: InVS15 (first row), Mid1 (second row), email-EU (third row), congress-bills (fourth row), house-committees (fifth row), music-reviews (sixth row). For each row, the committed nodes are selected through: the random, the top k-coreness, the top s-coreness, the top frequency-based R_w-hyper-coreness and the top size-independent R-hyper-coreness seeding strategies. The minority take-over, i.e. $n_A^* = 1$, takes place over an area A of the explored parameter space: its value, for each strategy, is reported in Supplementary Table IX. All simulations are run until the absorbing state with $n_A^* = 1$ is reached or the dynamics has evolved for t_{max} time steps and the stationary fraction n_A^* is obtained by averaging over 100 values sampled in the last T time-steps (see Supplementary Table VIII for the t_{max} and T values for each data set). The results refer to the median values obtained over 200 simulations. Cross markers in the heatmaps indicate the (β, p) values considered for Supplementary Fig. 39.

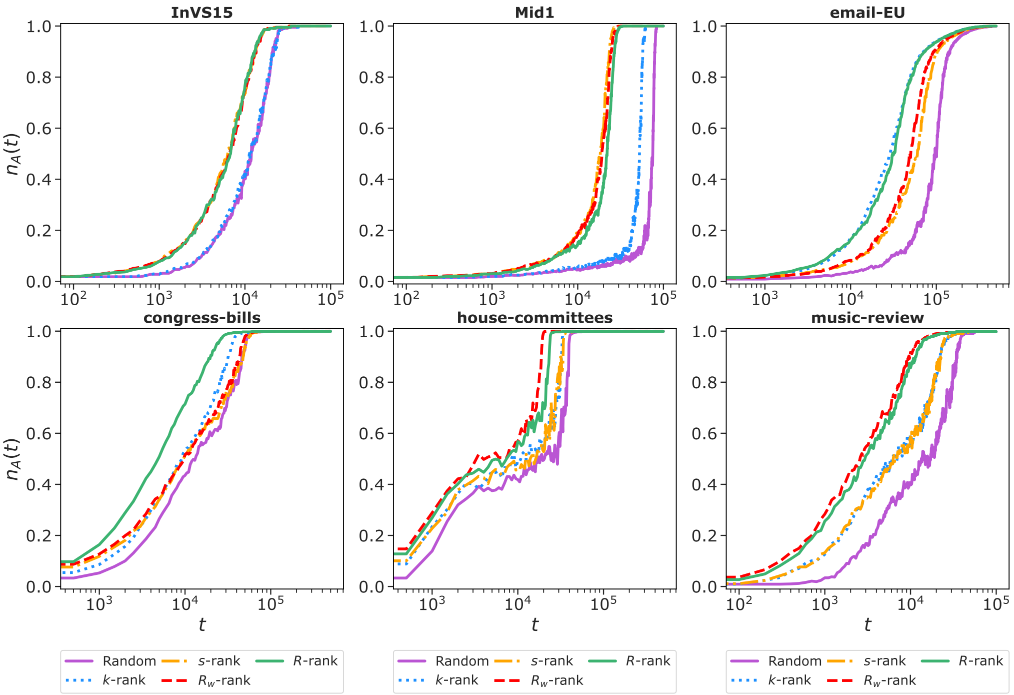

Supplementary Figure 38: **Higher-order NG process - Union rule - Temporal dynamics.** All panels show the temporal evolution of the fraction of nodes supporting only the name A, $n_A(t)$, according to the different seeding strategies for the committed minority and for fixed values of the agreement probability β and of the fraction of committed nodes p (see the cross markers in the heatmaps of Supplementary Fig. 36 and the parameters values in Supplementary Table VI). We consider the union rule and the following data sets: InVS15, Mid1, email-EU, congress-bills, house-committees, music-reviews. All results are obtained in the same simulation conditions of Fig. 36.

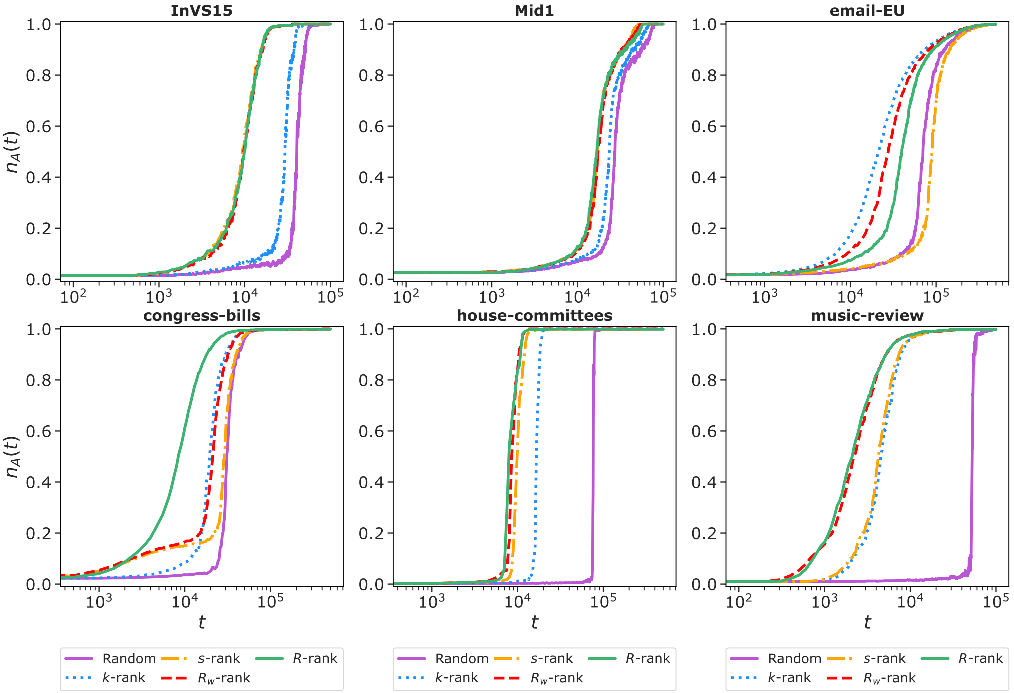

Supplementary Figure 39: **Higher-order NG process - Unanimity rule - Temporal dynamics.** All panels show the temporal evolution of the fraction of nodes supporting only the name A, $n_A(t)$, according to the different seeding strategies for the committed minority and for fixed values of the agreement probability β and of the fraction of committed nodes p (see the cross markers in the heatmaps of Supplementary Fig. 37 and the parameters values in Supplementary Table VIII). We consider the unanimity rule and the following data sets: InVS15, Mid1, email-EU, congress-bills, house-committees, music-reviews. All results are obtained in the same simulation conditions of Fig. 37.

VII. SUPPLEMENTARY NOTE 7: DESCRIPTION OF THE HYPER-CORE DECOMPOSITION PROCEDURE

Given a static hypergraph $\mathcal{H}$ composed of N nodes connected by E hyperedges of different sizes $m \in [2, M]$, the strightforward implementation of the hyper-core decomposition procedure to identify the (k, m)-core is performed as follows:

1. we consider the subhypergraph $\mathcal{H}_m \subseteq \mathcal{H}$ containing only the E_m hyperedges of size at least m;

2. for each node i, we calculate the total degree $D_m(i)$ in $\mathcal{H}_m$, i.e. the total number of hyperedges in which the node i is involved in $\mathcal{H}_m$;

3. all nodes with degree D_m lower than k are removed from $\mathcal{H}_m$ and from the hyperedges in which they are involved, thus reducing their size. In this way, the subhypergraph $\mathcal{H}'_m$ is obtained;

4. any hyperedges in $\mathcal{H}'_m$ of size lower then m, obtained by the nodes removal, are removed obtaining the subhypergraph $\mathcal{H}''_m$;

5. any fully coincident hyperedges in $\mathcal{H}''_m$, generated by the nodes removal, are removed so that each hyperedge appears only once, thus obtaining the subhypergraph $\mathcal{H}'''_m$;

6. the procedure is repeated iteratively from point 2, considering $\mathcal{H}_m = \mathcal{H}'''_m$, until all the nodes in $\mathcal{H}'''_m$ have degree D_m at least k and in $\mathcal{H}'''_m$ there are only not-fully-coincident hyperedges of size at least m.

To obtain the complete hyper-core structure, i.e. all the (k, m)-cores, the procedure is repeated for each $k \in [1, k^m_{max}]$ and for each size $m \in [2, M]$.

Note that the procedure is analogous to the k-core decomposition on static graphs [7, 8]: point 1 corresponds to considering all the links in the graph, and point 4 corresponds to removing from the graph all the links in which the removed nodes are involved. However, there are two main differences:

- when a node is removed, in the k-core decomposition procedure on a graph, all the links in which it is involved are removed; on the contrary in the (k, m)-core decomposition a hyperedge of size m' in which the node is involved remains but representing an interaction of lower size $m'' < m'$. This change in the size of the hyperedges requires to search and remove from the new hypergraph $\mathcal{H}'_m$ any hyperedge whose new size is lower then m or fully coincident hyperedges (points 4-5);

- the decomposition procedure is repeated for each order of interaction $m \in [2, M]$.

The algorithmic complexity of the k-core decomposition of a graph is $\mathcal{O}(N + E)$ [7, 8]; similarly the algorithmic complexity of the described implementation of the (k, m)-core decomposition is $\mathcal{O}(M[N + E \log(E)])$, where the new terms M and $\log(E)$ are due to the two differences described above. We show this scaling in Supplementary Fig. 40.

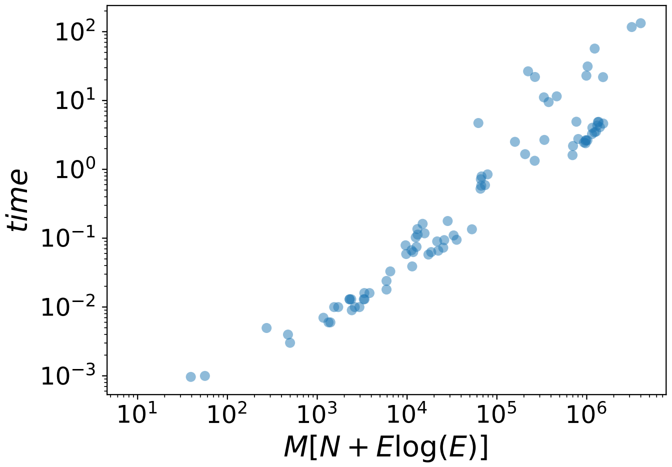

Supplementary Figure 40: **Time complexity of the (k, m)-core decomposition procedure.** We show the computation time for obtaining the (k, m)-core structure as a function of $x = M[N + Elog(E)]$ for some of the considered data sets. To have a larger statistics and variability in terms of N, M and E, we build additional hypergraphs by considering separately the interactions in specific time windows for the data sets where temporal information is available, aggregating the face-to-face interactions data (SocioPatterns and Utah Schools) over each day, email-EU data over each month and email-Enron data over each bimester. We then perform the (k, m)-core decomposition on each obtained static hypergraphs. A linear regression yield $\log(t) \sim a \log(x)$, with $a = 1.01 \pm 0.04$, with a Pearson correlation coefficient $r = 0.95$ and a p-value $p \ll 0.001$.

SUPPLEMENTARY REFERENCES

[1] N. W. Landry and J. G. Restrepo, Hypergraph assortativity: A dynamical systems perspective, Chaos: An Interdisciplinary Journal of Nonlinear Science **32**, 053113 (2022).
[2] I. Malvestio, A. Cardillo, and N. Masuda, Interplay between k-core and community structure in complex networks, Scientific Reports **10**, 14702 (2020).
[3] G. St-Onge, I. Iacopini, V. Latora, A. Barrat, G. Petri, A. Allard, and L. Hébert-Dufresne, Influential groups for seeding and sustaining nonlinear contagion in heterogeneous hypergraphs, Communications Physics **5**, 25 (2022).
[4] G. Ferraz de Arruda, G. Petri, P. M. Rodriguez, and Y. Moreno, Multistability, intermittency, and hybrid transitions in social contagion models on hypergraphs, Nature Communications **14**, 1375 (2023).
[5] I. Iacopini, G. Petri, A. Baronchelli, and A. Barrat, Group interactions modulate critical mass dynamics in social convention, Communications Physics **5**, 64 (2022).
[6] G. F. de Arruda, G. Petri, and Y. Moreno, Social contagion models on hypergraphs, Phys. Rev. Research **2**, 023032 (2020).
[7] J. I. Alvarez-Hamelin, L. Dall'Asta, A. Barrat, and A. Vespignani, K-core decomposition of internet graphs: hierarchies, self-similarity and measurement biases, Networks and Heterogeneous Media **3**, 371 (2008).
[8] V. Batagelj and M. Zaversnik, An o(m) algorithm for cores decomposition of networks, arXiv preprint - arXiv:cs/0310049 (2003).
